# Efficacy of acupuncture-related treatment for sleep disturbances in children with neurodevelopmental disorders: a systematic review and meta-analysis

**DOI:** 10.3389/fpsyt.2025.1670438

**Published:** 2026-01-13

**Authors:** Yoon Kyoung Jeong, Sun Haeng Lee

**Affiliations:** 1 Sungshin Hospital of Korean Medicine, Seongnam-si, Republic of Korea; 2 Department of Korean Pediatrics, Graduate School, Kyung Hee University, Seoul, Republic of Korea; 3 Department of Korean Pediatrics, College of Korean Medicine, Kyung Hee University, Kyung Hee University Medical Center, Seoul, Republic of Korea

**Keywords:** acupuncture, meta-analysis, neurodevelopmental disorders, nighttime awakenings, sleep anxiety, sleep disturbances

## Abstract

**Aim:**

To evaluate the efficacy of acupuncture-related treatments for sleep disturbances in children with neurodevelopmental disorders (NDDs).

**Methods:**

A search of 16 databases on May 3, 2025, identified randomized controlled trials on acupuncture-related treatments for this population. The primary outcome was the total score of the Children’s Sleep Habits Questionnaire (CSHQ), with secondary outcomes including CSHQ subscale scores, total effective rate (TER), and polysomnographic parameters. A meta-analysis using R Studio 4.4 included subgroup analyses by acupuncture modality. The risk of bias was evaluated using the Cochrane tool, and the quality of evidence was evaluated.

**Results:**

Seventeen studies involving 1,091 children were included. Acupuncture-related treatment combined with conventional treatment (CT) significantly reduced the CSHQ total scores compared to CT alone (p < 0.0001), with notable improvements in sleep anxiety (p = 0.0023) and nighttime awakenings (p < 0.0001). Improvements were also observed in TER and polysomnographic parameters, such as nighttime sleep time and awakening, with few adverse events. The risk of publication bias was low, and the quality of evidence was rated as moderate for the CSHQ total score and TER.

**Interpretation:**

Acupuncture-related treatment may be an effective adjunct to nonpharmacological interventions for enhancing sleep disturbances in children with NDDs.

**Systematic review registration:**

https://www.crd.york.ac.uk/PROSPERO/view/CRD42024545366, identifier CRD42024545366.

## 1 Introduction

Sleep disturbances are common among children and adolescents with neurodevelopmental disorders (NDDs), affecting up to 80% of the population (1). Sleep problems have frequently been reported in children with autism spectrum disorder (ASD) (32%–71.5%), attention-deficit/hyperactivity disorder (ADHD) (25%–50%), cerebral palsy (23%–46%), and intellectual disability (up to 86%) (1, 2). Sleep problems in children with NDDs exacerbate daytime fatigue and poor emotional regulation (3, 4), contributing to mental health problems such as anxiety, depression, and aggression (5). Therefore, sleep problems and emotional dysregulation are now recognized as correlated symptoms of NDDs rather than as independent comorbidities (6).

NDDs are conditions caused by the early impairments of brain development. These conditions include intellectual disability, ASD, ADHD, specific learning disorder, motor disorder such as tic, developmental coordination disorder, stereotypic movement disorder, and cerebral palsy (7, 8). In the United States, approximately 15% of children and adolescents are diagnosed with NDDs (9).

Despite their clinical importance, pharmacological treatment options for sleep disturbances in children with NDDs remain limited (10), and non-pharmacological interventions such as cognitive behavioral therapy and sleep hygiene are recommended as first-line treatments (11). Reflecting on this clinical situation, 29% of children with sleep problems in the United States use at least one form of complementary and alternative therapy (12). Among these therapies, acupuncture has emerged as a promising option for pediatric NDDs (7) as it is thought to enhance sleep by regulating the balance between the autonomic nervous system and neurotransmitter levels (13). Moreover, previous studies have shown that acupuncture has a favorable safety profile with few side effects (14, 15) and no risk of tolerance or dependence.

However, no meta-analysis has specifically evaluated the efficacy of acupuncture-related treatments for sleep disturbances in children with NDDs. Previous meta-analyses have focused on the individual symptoms, particularly ADHD, ASD, and Tourette syndrome (14–17), rather than on sleep disturbances. Therefore, this review aimed to systematically evaluate the efficacy of acupuncture-related treatments for sleep disturbances in children with NDDs and to support their potential as effective non-pharmacological options.

## 2 Methods

### 2.1 Protocol registration

The research protocol was registered on the PROSPERO platform before the start of the study (https://www.crd.york.ac.uk/PROSPERO/view/CRD42024545366). As this was a systematic review and secondary analysis of existing research, no patient informed consent or ethical approval was required.

### 2.2 Eligibility criteria

#### 2.2.1 Study design

Only parallel-group randomized controlled trials (RCTs) evaluating the efficacy of acupuncture-related treatments were included.

#### 2.2.2 Participants

We included studies that enrolled children and adolescents under the age of 18 years who were diagnosed with NDDs such as intellectual disability, ASD, ADHD, specific learning disorder, motor disorder, developmental coordination disorder, stereotypic movement disorder, and cerebral palsy. These participants had comorbid sleep disturbances diagnosed based on clearly defined criteria. There were no restrictions on participants’ nationality, sex, duration of sleep disturbance, or type of NDD. Studies involving adult participants or non-NDD populations were excluded.

#### 2.2.3 Interventions and comparisons

The treatment group received acupuncture-related treatment in combination with conventional treatment (CT), whereas the control group received CT alone, including medication, behavioral therapy, sensory integration training, and sleep hygiene. Acupuncture-related treatments included all modalities involving the stimulation of acupoints, such as manual acupuncture, electroacupuncture, intradermal acupuncture (e.g., press needle), moxibustion—a traditional East Asian therapy in which the dried herb mugwort (*Artemisia vulgaris*) is applied to acupoints and burned to produce a warming effect—and acupressure. Studies that did not include acupuncture-related treatment as an intervention or provide acupoint information were excluded. No limit to the number of interventions was imposed (e.g., alone or in combination). The treatment group received the same CTs as the control group. Studies in which the control group received an acupuncture-related treatment were excluded.

#### 2.2.4 Outcome measures

The primary outcome was the post-treatment total score of the Children’s Sleep Habits Questionnaire (CSHQ), a parent-report instrument originally developed to screen for sleep disturbances in children aged 4–10 years (18). The CSHQ has been validated in children with ASD and is widely employed in studies involving other NDDs, including ADHD and intellectual disability. Although optimal thresholds may vary by population, a total score above 41 is commonly used as a clinical cutoff for sleep disturbance. This study included both the original 8-subscale version and the shortened 5-subscale version adapted for Chinese children aged 3–5 years (19).

Secondary outcomes included CSHQ subscale scores, total effective rate (TER), and polysomnography (PSG) parameters. The TER indicates treatment efficacy based on CSHQ score and sleep-related clinical symptoms and is divided into four levels: “cured” (N1), “significantly effective” (N2), “effective” (N3), and “ineffective” (N4). This was calculated using the following formula: {(N1 + N2 + N3)/total number of cases} × 100 (%).

### 2.3 Information sources and search strategy

Sixteen databases were searched for relevant studies. All studies were searched without restrictions on the country, language, or year of publication. The original literature search was conducted on May 27, 2024, using a combination of medical subject headings (MeSH terms) and keywords related to NDD and sleep disturbance. An updated search was conducted on May 3, 2025, to ensure and reflect the most recent evidence. The search strategies were reviewed by experts in the field of pediatrics in Korean medicine and are presented in **Supplementary Table S1**. The following databases were used in the search:

1. English databases: PubMed, Elton B. Stephens Company (EBSCO), Embase, the Cochrane Central Register of Controlled Trials (CENTRAL), the Allied and Complementary Medicine Database (AMED), the Scientific Abstract and Citation Database (SCOPUS), the Web of Science, the International Clinical Trials Registry Platform (ICTRP), and ClinicalTrials.gov.
2. Chinese databases: China National Knowledge Infrastructure (CNKI), the Chinese Science and Technology Periodic Database (VIP), and the Wanfang Database.
3. Korean databases: the Oriental Medicine Advanced Search Integrated System (OASIS), Korean studies Information Service System (KISS), and the Korean Medical Database (KMbase).
4. Japanese database: Citation Information by the National Institute of Informatics (CiNii).

### 2.4 Study selection and data extraction

Two researchers independently searched the databases and exported the results to Endnote 21, a bibliographic information management software (Clarivate Analytics, Philadelphia, PA, USA). After removing duplicates, studies that were not relevant to this search were excluded based on their titles and abstracts. A full-text review was conducted to select the RCTs that satisfied the inclusion criteria. Each process was carefully verified through cross-checking, and disagreements were resolved through a discussion.

The characteristics of the selected studies were summarized in detail by two researchers and extracted using an Excel 2024 spreadsheet. The extracted data included study characteristics (first author’s name, year of publication, country, study setting, and funding), participant characteristics (sample size, age, sex, type of NDD, diagnostic criteria for sleep disturbance, and duration of sleep disturbances), intervention characteristics (acupuncture modality, acupoints, number of needles, depth of insertion, needle stimulation, needle retention time, treatment frequency, and duration), and clinical outcomes. For missing clinical outcome data, the corresponding authors were contacted via e-mail. If no response was received after two attempts, we proceeded with the analyses using the available data. The acupuncture-related treatment regimens were evaluated using the Standards for Reporting Interventions in Clinical Trials of Acupuncture (STRICTA) (20) (**Supplementary Table S2**).

### 2.5 Risk of bias assessment

Two researchers independently assessed the risk of bias using the revised Cochrane Risk of Bias Assessment tool (ROB 2) (21). The evaluation encompassed six domains: randomization process, deviations from intended interventions, missing outcome data, measurement of outcomes, selection of the reported result, and overall risk of bias. Each domain was rated as “low,” “some concern,” or “high” based on predefined criteria. Disagreements were resolved through discussion.

### 2.6 Statistical analysis

The meta-analysis was conducted using the R program 4.4.3 (R Foundation for Statistical Computing, Vienna, Austria) and the “meta” package. Dichotomous data were analyzed using the risk ratio (RR) with a 95% confidence interval (CI), whereas continuous data were synthesized using either the mean difference (MD) or standardized mean difference (SMD). MD was used when the measurement scales were identical; otherwise, SMD was applied. Although studies that included medication (e.g., pharmacologic agents) as part of CT were eligible, only those that included CT without medication were included in the meta-analysis to reduce heterogeneity and ensure comparability across studies.

Statistical heterogeneity was evaluated using Higgins’ I² statistics and p-values, whereas clinical heterogeneity was evaluated based on variations in acupuncture-related interventions (e.g., acupuncture, acupressure, press needle, and moxibustion). A random-effects model was applied to account for clinical heterogeneity, given the diversity of both NDDs and acupuncture-related interventions across the studies. When substantial heterogeneity (I² ≥50%) was detected, subgroup analysis was conducted based on the type of acupuncture-related intervention. As the number of studies within each subgroup was small, a meta-analysis of variance was performed with equal variance across subgroups.

Publication bias was visually examined using a funnel plot of outcomes that included more than 10 studies. Funnel plot asymmetry was evaluated using the Egger’s regression test (22). Sensitivity analyses were conducted using a leave-one-out approach in which each study was sequentially excluded to examine its influence on the overall estimate.

### 2.7 Quality of evidence

The quality of evidence was assessed using the Grading of Recommendations Assessment, Development, and Evaluation (GRADE) approach (23). Each outcome was evaluated for the following domains: risk of bias, inconsistency, indirectness, imprecision, and publication bias. For inconsistency, the certainty of evidence was downgraded by one level if the statistical heterogeneity was substantial (I^2^ between 50% and 75%) and the direction of the effect was consistent. For imprecision, the evidence was downgraded if the total sample size for a continuous variable was <300, or if the 95% CI of the pooled effect estimate included the null line and the minimal important difference (e.g., MD ± 0.5).

## 3 Results

### 3.1 Study selection

**Figure 1:**
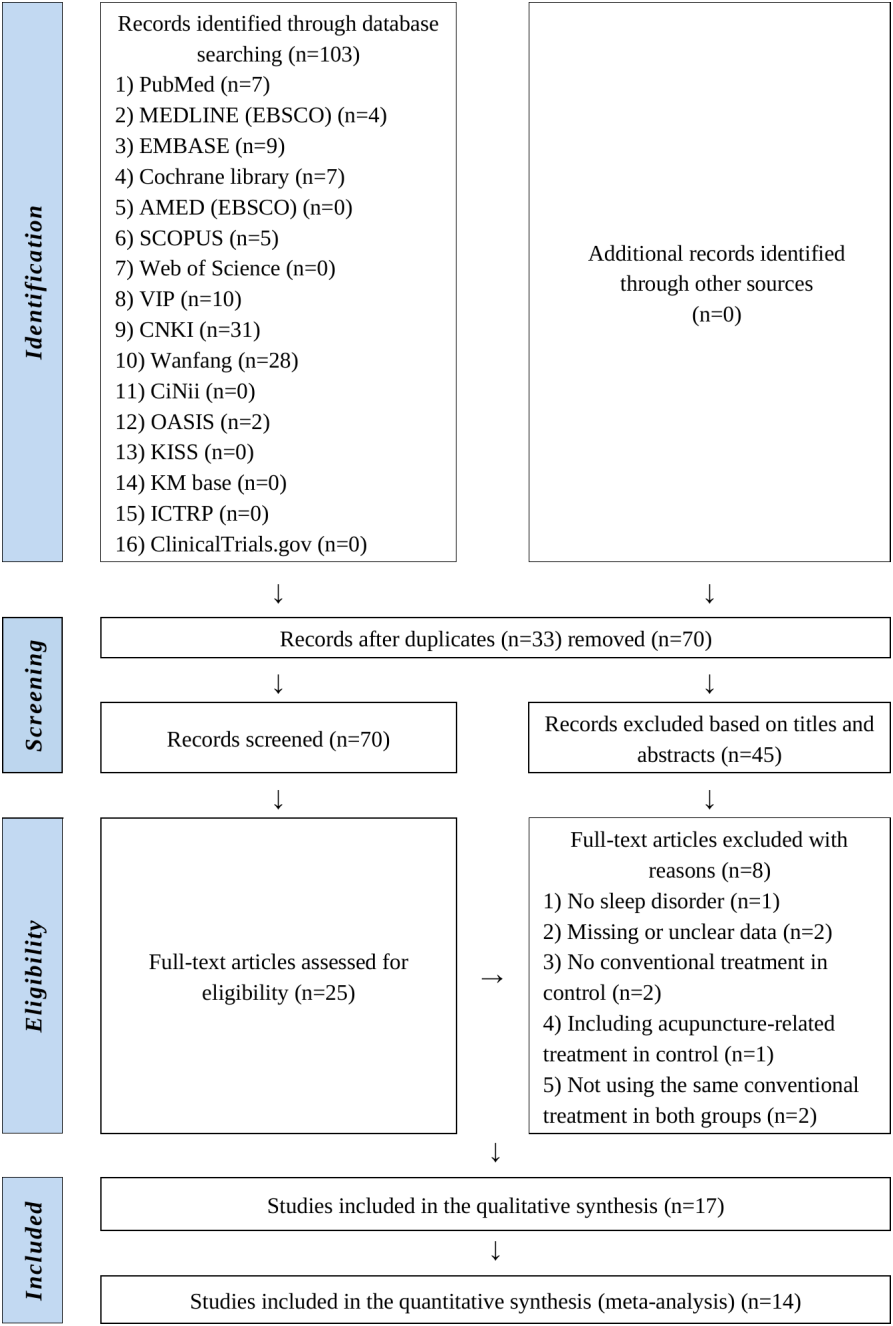
PRISMA flowchart of the literature selection process. PRISMA, Preferred Reporting Items for Systematic Reviews and Meta-Analyses.

A total of 103 relevant studies were identified from the 16 databases. After eliminating 33 duplicates, the remaining 70 studies underwent initial screening based on titles and abstracts, after which 45 studies were excluded. Full-text reviews were conducted for the remaining 25 studies, leading to the further exclusion of one that did not address sleep disturbances, two with missing or unclear outcome data, two lacking CTs in the control group, one including acupuncture-related intervention in the control group, and two with inconsistent CTs between groups, leaving 17 studies (24–40) which satisfied the eligibility criteria. Fourteen studies were included in the meta-analysis. A flowchart of the Preferred Reporting Items for Systematic Reviews and Meta-Analyses (PRISMA) (41) is illustrated in **Figure 1**.

### 3.2 Study characteristics

#### 3.2.1 Basic information of the included studies

Detailed characteristics of the 17 selected studies are summarized in **Supplementary Table S3**. Sixteen studies were conducted in Chinese hospitals, and one was conducted in an Iranian hospital. All the articles were published between 2014 and 2025. Eleven studies (24, 25, 29–31, 34–38, 40) were retrieved from CNKI, five (26–28, 32, 33) from the Wanfang database, and one (39) from Pubmed. Twelve studies reported funding sources, whereas five (28, 30, 32, 33, 37) did not provide information. This review included 1,091 children with NDDs accompanied by sleep disturbance. No significant differences were observed in baseline characteristics (e.g., mean age, sex, and CSHQ scores) between the treatment and control groups in any of the included studies.

#### 3.2.2 Underlying NDDs

ASD was the most frequently reported condition, appearing in nine studies (26, 27, 29, 31, 32, 34–36, 38). Cerebral palsy was reported in six studies, including two on spastic cerebral palsy (25, 40) and four without a detailed classification (24, 28, 30, 33). ADHD (39) and intellectual disability (37) were reported in one study. Only two studies have described the severity of this condition: one (34) for ASD and one (37) on intellectual disability.

#### 3.2.3 Diagnostic criteria for sleep disturbances

All studies described clear diagnostic criteria for sleep disturbance. Eleven used the CSHQ total score with varying cutoffs: four applied ≥54 points (29, 32, 35, 36), five used 41 points (27, 31, 34, 39, 40), while one study each used 48 points (33) and 45 points (25). Three studies (25, 28, 38) used the Chinese Classification of Mental Disorders (CCMD-3), while one study each used the International Classification of Sleep Disorders (24), the Diagnostic and Statistical Manual of Mental Disorders (DSM 5^th^ edition) (26), PSG (37), and the Encyclopedia of Mongolian Studies (30) for diagnosis. One study simultaneously applied the CSHQ and CCMD-3 criteria (25) or the CSHQ and ICD-11 criteria (40).

#### 3.2.4 Type of intervention models

Sixteen studies had a two-arm design, while one (24) had a three-arm design (only eligible arms that satisfied the inclusion criteria were analyzed). All treatment and control groups received CTs. Fourteen studies compared acupuncture-related treatment combined with CT without medication to CT without medication alone. The most common comparison was acupuncture plus CT versus CT in five studies (24, 27, 31, 32, 37), followed by press needle plus CT versus CT in three studies (29, 35, 36), moxibustion plus CT versus CT in two studies (25, 30), acupuncture plus acupressure plus CT versus CT in two studies (26, 28), acupuncture plus press needle plus CT versus CT in one study (34), and transcutaneous electrical acupoint stimulation plus CT versus CT in one study (38).

Two studies (33, 40) compared acupuncture-related treatment combined with CT, including medication, with the same CT alone, whereas one study (39) compared acupuncture-related treatment and medication with sham acupuncture and medication.

#### 3.2.5 Type of outcome measurements

Fourteen studies (25–29, 31–36, 38–40) reported CSHQ total scores, of which 10 also provided individual subscale scores. Eleven (25–28, 31, 33–35, 38–40) used the original 8-subscale version of the CSHQ, whereas three (29, 32, 36) used the shortened 5-subscale version. Furthermore, 10 studies assessed TER, and four reported objective sleep parameters assessed by PSG, including the number of nighttime awakenings, sleep efficiency, total sleep time, sleep latency, and REM sleep duration. One study (40) reported on the Pittsburgh Sleep Quality Index.

#### 3.2.6 Acupuncture regimen description

The acupuncture-related treatments included manual acupuncture, auricular press needles, transcutaneous electrical acupoint stimulation, acupressure, and moxibustion.

The duration, frequency, and retention time of acupuncture-related treatments varied across studies. The treatment duration ranged from 2 weeks to 6 months, with 12 weeks being the most common duration in seven studies (24, 26, 27, 32, 34, 37, 40). The frequency of treatments ranged from three to seven times per week, with three times per week being the most common, as reported in seven studies (24, 26, 28, 31, 32, 34, 37). The most common retention time per session was 30 minutes in 10 studies (24, 26, 28, 30–33, 37, 38, 40) (**Supplementary Tables S3** and **S4**). Most items were well reported in the STRICTA assessment; however, details on treatment variability, number of needles inserted, response sought, and practitioner background were often lacking (**Supplementary Table S2**).

#### 3.2.7 Frequently reported acupuncture points

The following 11 acupoints were identified as the most frequently reported for sleep disturbances in children with NDDs: Baihui (GV20) (24, 26, 30, 31, 33, 37, 38), Shenting (GV24) (24, 25, 28, 33, 34, 38, 40) (seven studies each, 41.18%), Anmian (EX-HN18) (24, 26, 28, 33, 37, 38), Shenmen (HT7) (24, 26, 28, 33, 37, 38), auricular Shenmen (TF4) (26, 28, 29, 35, 36, 39), auricular Heart (CO15) (26, 28, 29, 35, 36, 39), auricular Subcortex (AT4) (26, 28, 29, 35, 36, 39) (six studies each, 35.29%), Sishencong (EX-HN1) (28, 33, 34, 37, 40), Yintang (EX-HN3) (26, 31, 34, 37, 38) (five studies each, 29.41%), Sanyinjiao (SP6) (26, 33, 37, 38), auricular Liver (CO12) (26, 29, 35, 36) (four studies each, 23.53%) (**Table 1**).

**Table 1:** Top 11 most frequently reported acupuncture points.

Number of studies (%)	Acupuncture point
7 (41.18)	Baihui (GV20), Shenting (GV24)
6 (35.29)	Anmian (EX-HN18) Shenmen (HT7) Shenmen (TF4) Heart (CO15) Subcortex (AT4)
5 (29.41)	Sishencong (EX-HN1) Yintang (EX-HN3)
4 (23.53)	Sanyinjiao (SP6) Liver (CO12)

### 3.3 Risk of bias assessment

**Figure 2:**
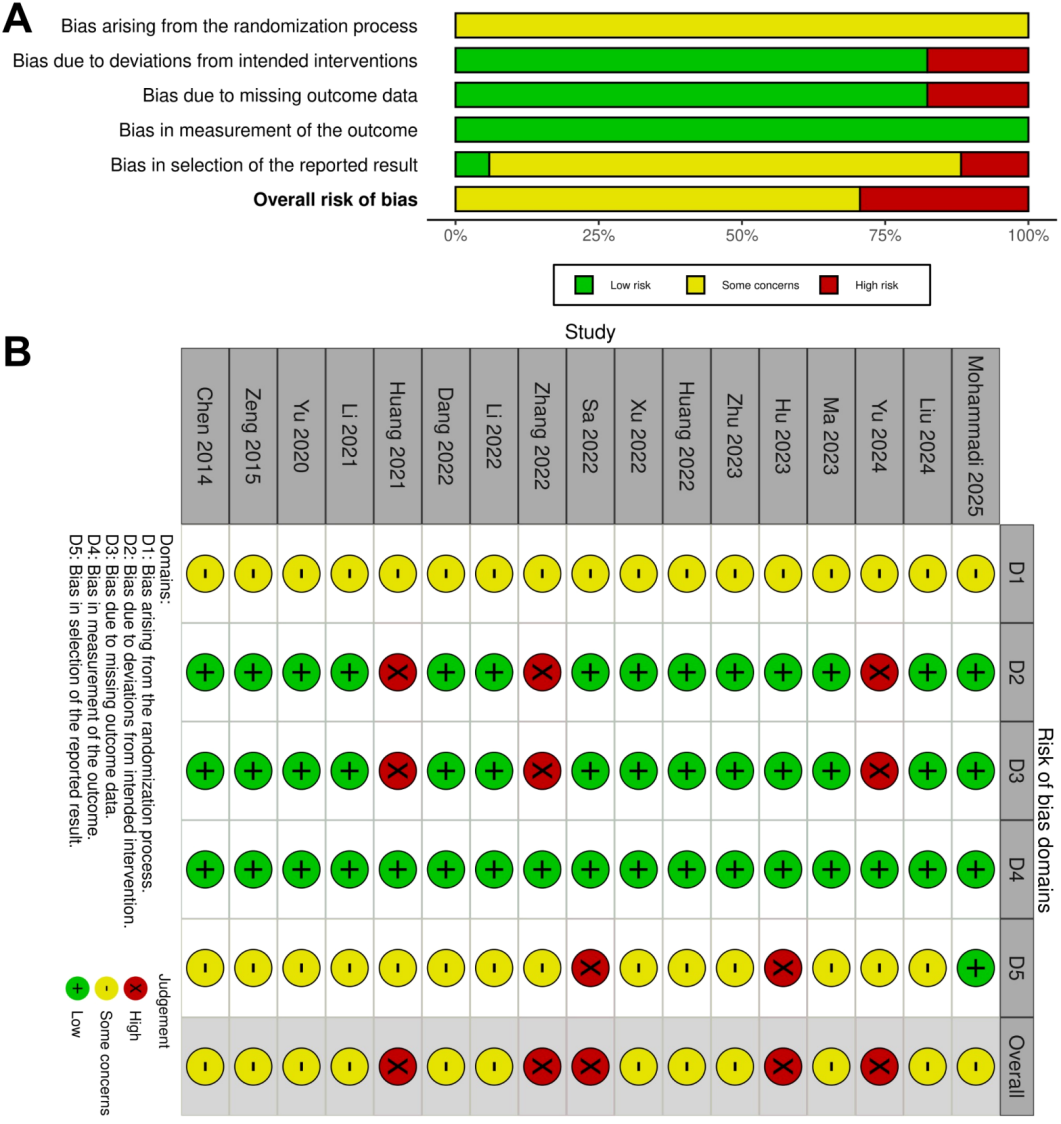
Risk of bias graph **(A)** and risk of bias summary **(B)**.

A total of 12 studies (25, 27–29, 32, 33, 35–40) were found to have some concerns, whereas five (24, 26, 30, 31, 34) were identified as having a high risk of bias. Three studies (24, 31, 34) lacked explanations for missing outcome data and did not define any criteria for adherence or participant exclusion, which resulted in a high risk of bias in the deviation domain. Two (26, 30) were identified as being at a high risk of selective reporting bias owing to the absence of pre-specified outcome measures. Although all studies mentioned random allocation, three (27, 30, 36) did not specify the randomization method. Only one study (39) reported information on the study protocol (**Figure 2**).

### 3.4 Meta-analysis results

#### 3.4.1 Primary outcomes

##### 3.4.1.1 CSHQ total score

**Figure 3:**
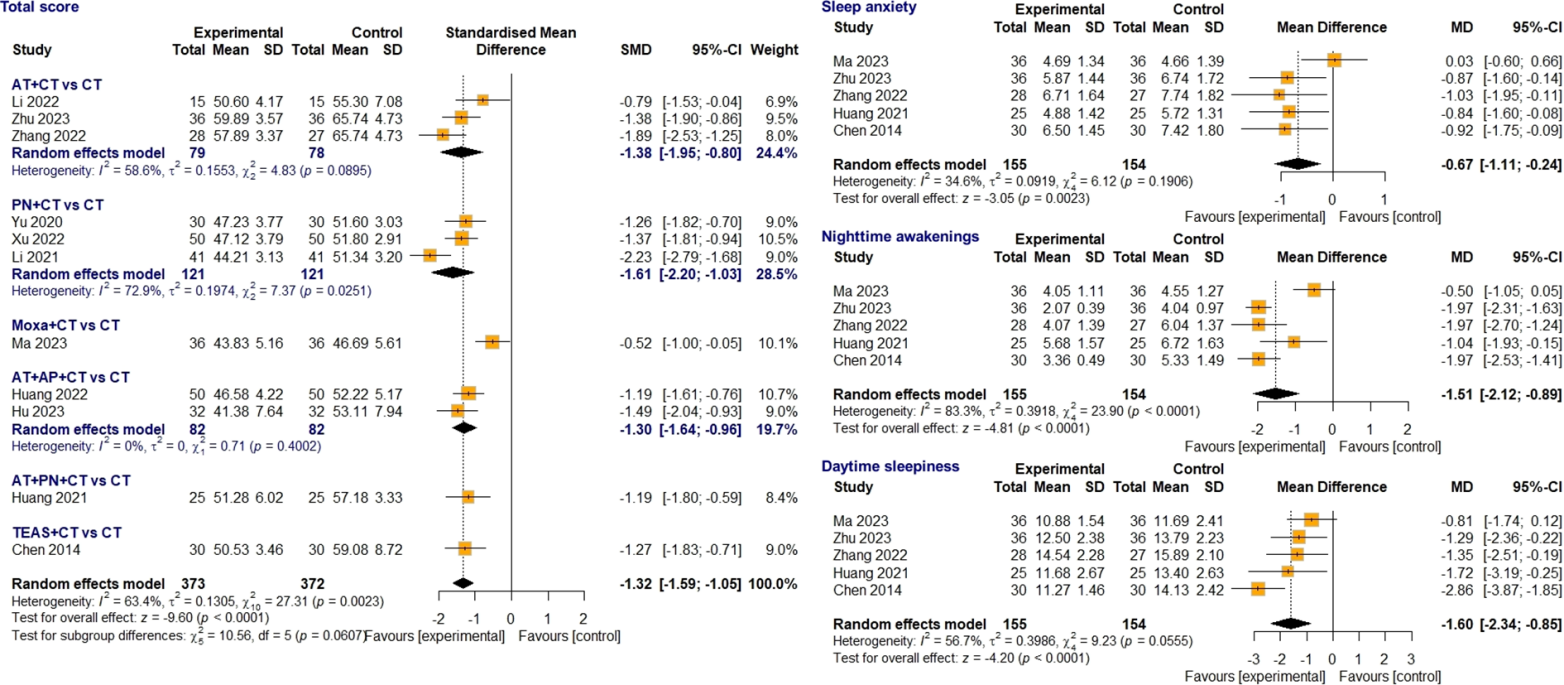
Forest plot comparing acupuncture-related treatment plus CT vs. CT on CSHQ total score and 8-subscale scores. AT, acupuncture; AP, acupressure; CT, conventional treatment; CSHQ, Children’s Sleep Habits Questionnaire; Moxa, moxibustion; PN, press needle; TEAS, transcutaneous electrical acupoint stimulation.

Among the 14 studies reporting the CSHQ total scores, 11 (25–29, 31, 32, 34–36, 38) were included in the meta-analysis. Because these studies used either the 8- or 5-subscale CSHQ, the effect size was calculated using SMD, showing high heterogeneity (I² = 63.4%, p = 0.002). A total of 373 and 372 children with sleep disturbances accompanying NDDs were included in both groups, respectively. The meta-analysis demonstrated that acupuncture-related treatment combined with non-pharmacological CT significantly decreased the CSHQ total score compared to CT alone, indicating improved sleep quality (SMD = -1.32, 95% CI [-1.59; -1.05], Z = -9.60, p < 0.0001) (**Figure 3**). Subgroup analyses based on the type of acupuncture-related treatment (e.g., acupuncture, press needle, moxibustion, transcutaneous electrical acupoint stimulation) did not reveal statistically significant differences. Acupuncture was used in three studies (27, 31, 32); press needle in three (29, 35, 36); acupuncture combined with auricular acupressure in two (26, 28); moxibustion in one (25); transcutaneous electrical acupoint stimulation in one (38); and acupuncture combined with press needle in one (34). Notably, the most significant improvements were observed in the subgroup that applied press needle at auricular acupoints, specifically the shenmen (TF4), heart (CO15), liver (CO12), and subcortex (AT4), once daily for 2–4 hours, in combination with CT. Moreover, one study (35) that showed the greatest effect in this subgroup, additionally stimulated the auricular acupoints of the lung (CO14), diaphragm (CO16), endocrine (CO18), and intestine (CO9).

#### 3.4.2 Secondary outcomes

##### 3.4.2.1 CSHQ 8-subscale scores

Among the seven studies that reported 8-subscale CSHQ scores, five (25, 27, 31, 34, 38) were included in the meta-analysis, with 155 children in the treatment group and 154 in the control group. The meta-analysis demonstrated statistically significant improvements in five subscales: sleep anxiety, nighttime awakening, daytime sleepiness, sleep duration, and sleep-disordered breathing. Daytime sleepiness was most significantly reduced after treatment (MD = -1.60, 95% CI [-2.34; -0.85], Z = -4.20, p < 0.0001), followed by nighttime awakenings (MD = -1.51, 95% CI [-2.12; -0.89], Z = -4.81, p < 0.0001), and sleep duration (MD = -0.94, 95% CI [-1.77; -0.11], Z = -2.23, p = 0.026). Sleep anxiety (MD = -0.67, 95% CI [-1.11; -0.24], Z = -3.05, p = 0.0023) and sleep-disordered breathing (MD = -0.30, 95% CI [-0.54; -0.07], Z = -2.55, p = 0.0107) improved with low heterogeneity. However, no statistically significant improvements were observed in bedtime resistance, sleep-onset delay, or parasomnias. Detailed results are presented in **Figure 3** and **Supplementary Figure S1**, highlighting the interconnections between core sleep subscales, particularly daytime sleepiness, nighttime awakening, and sleep anxiety, which are key psychological factors contributing to sleep disturbance.

##### 3.4.2.2 CSHQ 5-subscale scores

Three studies (29, 32, 36) reported 5-subscale scores that were abbreviated version of the CSHQ (19). The meta-analysis revealed statistically significant improvements in most subscales: sleep behavior (MD = -2.40, 95% CI [-4.64; -0.15], Z = -2.09, p = 0.036), daytime sleepiness (MD = -0.89, 95% CI [-1.61; -0.16], Z = -2.39, p = 0.017), bedtime habits (MD = -0.75, 95% CI [-1.30; -0.19], Z = -2.62, p = 0.009), and morning waking habits (MD = -0.65, 95% CI [-1.26; -0.03], Z = -2.07, p = 0.039) (**Supplementary Figure S2**). However, nighttime awakening demonstrated no significant improvement (SMD = -0.02, 95% CI [-0.30; 0.27], Z = -0.13, p = 0.899).

##### 3.4.2.3 TER

**Figure 4:**
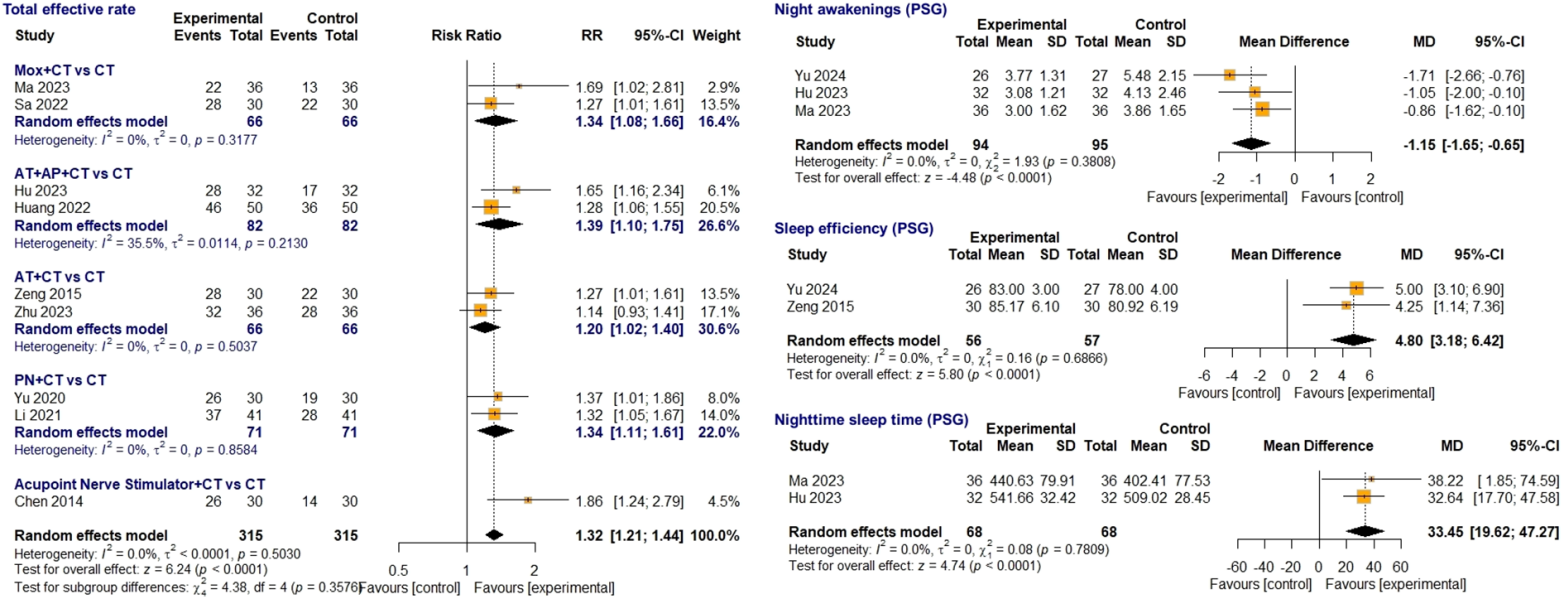
Forest plot comparing acupuncture-related treatment plus CT vs. CT on TER and PSG sleep parameters. AT, acupuncture; AP, acupressure; CT, conventional treatment; TER, total effective rate; Moxa, moxibustion; PN, press needle; PSG, polysomnography.

Among the 10 studies reporting TER, nine (25–28, 30, 35–38) were included in the meta-analysis, excluding one (33) that involved medication in CT. This meta-analysis included 315 children with sleep disturbances in both groups. Despite minimal statistical heterogeneity (I² = 0.0%, p = 0.503), we employed a random-effects model to account for clinical heterogeneity due to different acupuncture-related interventions. The treatment group revealed a statistically significant effect (RR = 1.32, 95% CI [1.21; 1.44], Z = 6.24, p < 0.0001). Acupuncture and auricular acupressure combined with CT (26, 28) showed the largest effect (RR = 1.39, 95% CI [1.10; 1.75]), followed by moxibustion with CT (25, 30), auricular press needle with CT (RR = 1.34, 95% CI [1.11; 1.61]) (35, 36), and acupuncture with CT (RR = 1.20, 95% CI [1.02; 1.40]) (27, 37) (**Figure 4**).

##### 3.4.2.4 PSG sleep parameters

Four studies (24–26, 37) assessed PSG parameters, including the number of nighttime awakenings, sleep efficiency, nighttime sleep time, sleep latency, and rapid eye movement (REM) sleep. The meta-analysis included three studies each on sleep latency (24, 25, 37) and the number of nighttime awakenings (24–26), and two studies on sleep efficiency (24, 37), REM sleep (26, 37), and nighttime sleep time (25, 26). Although minimal heterogeneity was detected (I² = 0.0%), a random-effects model was used to account for clinical heterogeneity. The acupuncture-related treatment group combined with CT demonstrated significantly reduced nighttime awakenings (MD = -1.15 times, 95% CI [-1.65; -0.65], Z = -4.48, p < 0.0001), increased sleep efficiency (MD = 4.80%, 95% CI [3.18; 6.42], Z = 5.80, p < 0.0001), and extended nighttime sleep time (MD = 33.45 minutes, 95% CI [19.62; 47.27], Z = 4.74, p < 0.0001) (**Figure 4**). However, no significant improvements were observed in sleep latency or REM sleep time.

#### 3.4.3 Other sleep parameters

The Pittsburgh Sleep Quality Index, as reported in one study (40), showed a significant improvement in the treatment group compared to the control group after treatment. Total sleep time (hours) (33), measured as a sleep-related metric in the CSHQ, significantly improved in the acupuncture group after treatment. Additionally, light and deep sleep time, as measured by PSG in one study (26), were significantly increased in the acupuncture group after treatment. However, the average sleep time (minutes), as assessed using the CSHQ in two studies (29, 32), revealed no statistically significant difference between the groups after treatment (p = 0.26) (**Supplementary Table S3**).

### 3.5 Adverse events

Four studies reported on AEs. Three studies (29, 32, 40) reported that no AEs occurred in either group, whereas one (36) reported a single case of local discomfort in the treatment group (**Supplementary Table S3**).

### 3.6 Publication bias

Publication bias was assessed based on the CSHQ total score, which included more than 10 studies, using Egger’s regression test and a funnel plot. The result showed low risk of publication bias (t = -0.60, df = 9, p = 0.5658) (**Supplementary Figure S3**).

### 3.7 Sensitivity analysis

Sensitivity analyses were conducted for all significantly improved outcomes, and all outcomes remained stable when the effect sizes were recalculated after excluding individual studies, except for bedtime and morning waking habits on the 5-subscale CSHQ (**Supplementary Figures S4A–N**).

### 3.8 Quality of evidence

The quality of evidence was assessed using the CSHQ total score and three core subscales (daytime sleepiness, nighttime awakenings, and sleep anxiety) of the original version of the CSHQ, as well as the TER and objective PSG parameters related to sleep maintenance and arousal. The quality of evidence for the CSHQ total score and TER was rated as “moderate.” However, the evidence for sleep anxiety and daytime sleepiness was downgraded to “low” because the confidence interval of one study included both the null value and the minimal important difference (e.g., MD ± 0.5). The evidence for nighttime awakening was also downgraded to “very low” due to the substantial heterogeneity. Although the studies that reported PSG parameters showed consistently significant effect sizes, the overall quality of the evidence was downgraded to “very low” due to the inclusion of studies with small sample sizes and high risk of bias (**Table 2**).

**Table 2:** Summary of findings of meta-analysis.

Outcomes	Anticipated absolute effects* (95% CI)		Relative effect (95% CI)	№ of participants (studies)	Certainty of the evidence (GRADE)
	Risk with CT	Risk with AT-related treatment plus CT			
CSHQ total score	–	SMD 1.32 SD lower (1.59 lower to 1.05 lower)	–	745 (11 RCTs)	⨁⨁⨁◯ Moderate^a,b^
Sleep anxiety (8-subscale CSHQ)		MD 0.67 lower (1.11 lower to 0.24 lower)		309 (5 RCTs)	⨁⨁◯◯ Low^a,c^
Daytime sleepiness (8-subscale CSHQ)		MD 1.6 lower (2.34 lower to 0.85 lower)		309 (5 RCTs)	⨁⨁◯◯ Low^a,b,c^
Nighttime awakenings (8-subscale CSHQ)		MD 1.51 lower (2.12 lower to 0.89 lower)		309 (5 RCTs)	⨁◯◯◯ Very low^a,c,d^
TER	632 per 1,000	865 per 1,000 (790 to 948)	RR 1.37 (1.21 to 1.44)	630 (9 RCTs)	⨁⨁⨁◯ Moderate^a,e^
Number of nighttime awakenings (PSG)	–	MD 1.15 times lower (1.65 lower to 0.65 lower)	–	189 (3 RCTs)	⨁◯◯◯ Very low^f,g^
Sleep efficiency (PSG)	–	MD 4.8% higher (3.18 higher to 6.42 higher)	–	113 (2 RCTs)	⨁◯◯◯ Very low^f,g^
Nighttime sleep time (PSG)	–	MD 33.45 minutes higher (19.62 higher to 47.27 higher)	–	136 (2 RCTs)	⨁◯◯◯ Very low^f,g^

GRADE, Grading of Recommendations Assessment, Development, and Evaluation; AT, acupuncture; CT, conventional treatment; RCT, randomized controlled trial; CSHQ, Children’s Sleep Habits Questionnaire; TER, total effective rate; PSG, polysomnography; CI, confidence interval; MD, mean difference; RR, risk ratio; SMD, standardized mean difference.

a. Most studies had some concerns about the risk of bias, but less than 50% had a high risk of bias.

b. Despite moderate heterogeneity (50%≤ I² <75%), the effect direction was consistent, and the CIs partially overlapped, resulting in no downgrading.

c. The 95% confidence intervals of some studies included null values and minimal important differences.

d. Despite substantial heterogeneity (I² ≥75%), the consistent effect direction resulted in downgrading by only one level.

e. Because the optimal information size (OIS) criterion was met, and the contribution of the study, including the null value, was minimal, downgrading was not warranted.

f. More than 50% of the included studies had a high risk of bias.

g. Sample size (continuous variable) < 300.

## 4 Discussion

### 4.1 Main findings

This review aimed to evaluate the efficacy and safety of acupuncture-related treatments for sleep disturbances in children and adolescents with NDDs. Despite the comprehensive search strategy encompassing all types of NDDs, the included studies focused on cerebral palsy, ASD, ADHD, and intellectual disability, and no RCTs were found for other NDDs such as tic disorder.

This study conducted a qualitative synthesis of seventeen RCTs comparing acupuncture-related treatment combined with CT with CT alone. After excluding three studies (33, 39, 40) that incorporated medication as part of CT, a total of 14 RCTs were included in the meta-analysis.

The results of meta-analysis indicated that the combination of acupuncture-related treatment and CT may enhance sleep quality, as shown by the reduction in the CSHQ total score (SMD -1.32, Z = -9.60, p <0.0001). Among the eight subscales of the original CSHQ, significant improvements were noted in sleep anxiety, nighttime awakenings, and daytime sleepiness. These three factors were closely correlated to emotional and arousal dysregulation in children with NDDs. These findings suggest that acupuncture-related treatment may have a beneficial effect on sleep quality in this population by addressing the underlying psychological factors that contribute to sleep disturbance.

Furthermore, the findings revealed consistent improvements in daytime sleepiness across both the 5- and 8-subscale versions of the CSHQ, indicating its potential effects on nighttime sleep quality and daytime functioning. Moreover, the TER, typically calculated based on the CSHQ score and clinical symptoms, was higher in the acupuncture-related treatment groups than in the control groups (RR 1.32, p <0.0001). In studies using PSG to assess sleep, acupuncture-related treatments revealed improvements in objective sleep parameters. The improvements included a reduction in the frequency of nighttime awakenings by a mean of 1.15 (p < 0.0001), enhancement in sleep efficiency by a mean of 4.8% (p < 0.0001), and increase in nighttime sleep time by an average of 33.45 minutes (p < 0.0001).

Although both the PSG and CSHQ outcomes revealed consistent improvements in overall sleep continuity and quality, these outcomes focus on disparate aspects of sleep. While the PSG captures objective sleep architecture during a single night, the CSHQ reflects parent-reported habitual sleep patterns over the past month (42). Therefore, given its broader scope and widespread use in pediatric populations, the CSHQ total score was selected as the primary outcome in this review.

### 4.2 Clinical implications

These findings have three key clinical implications and support the integration of acupuncture-related treatments into existing non-pharmacological approaches to address sleep disturbances in children with NDDs.

First, when combined with conventional non-pharmacological treatment, acupuncture-related treatments demonstrated the potential of improving both sleep quality and quantity, as indicated by reduced nighttime awakenings and increased sleep efficiency and total sleep time, as measured using both the CSHQ and PSG.

Second, acupuncture-related treatments may help alleviate the psychological factors that interfere with sleep, thereby making bedtime more manageable. Particularly, sleep anxiety was significantly reduced, indicating the potential benefits of stabilizing sleep architecture and emotional regulation.

Third, by reducing daytime sleepiness, acupuncture-related treatments may improve daytime functioning and ultimately enhance overall daily activity levels compared to CT alone.

### 4.3 Frequently reported acupuncture points

The acupoints most frequently used for sleep disturbances in children diagnosed with NDDs were GV20, GV24, EX-HN18, HT7, TF4, CO15, AT4, EX-HN1, EX-HN3, SP6, and CO12. Among these acupoints, GV20 has been extensively studied for its role in neuropsychiatric disorders, with evidence suggesting an increase in α wave frequency related to relaxation and a decrease in β wave frequency associated with stress and anxiety, thereby contributing to the suppression of cerebral excitability (43). Governor vessel acupoints such as GV20 and GV24 have frequently been selected for the treatment of insomnia (44). Similarly, HT7 modulates the hypothalamic-pituitary-adrenal axis and lowers stress hormone levels, thereby alleviating anxiety (45). Electroacupuncture at HT7 also increases the levels of brain-derived neurotrophic factors and enhances stress resilience, thereby contributing to improved sleep (46). Furthermore, clinical studies have suggested that acupuncture involving GV20 (47) and HT7 (47) may be superior to conventional sleep medications including trazodone, for improving sleep quality and daytime function. These findings support the frequent use of GV20 and HT7 and highlight their potential neurophysiological mechanisms for treating sleep disorder.

### 4.4 Neurophysiological mechanisms of acupuncture in sleep regulation

The improvements in sleep continuity and quality observed in this review are closely associated with the autonomic nervous system and neurotransmitter regulation in acupuncture (48). Children diagnosed with NDDs frequently experience hyperarousal and sleep disturbances that are attributable to autonomic dysfunction (49, 50). Acupuncture helps to restore the autonomic balance by reducing sympathetic hyperactivity and promoting parasympathetic activity (51), thereby alleviating sleep anxiety (13).

This review showed that acupuncture-related treatments reduced sleep anxiety and nighttime awakening. Patients with insomnia often exhibit decreased γ-aminobutyric acid (GABA) levels, leading to cerebral cortical excitability and difficulty in maintaining sleep (52). Previous studies have reported that acupuncture may reduce brain arousal and regulate sleep by increasing serotonin, melatonin, and GABA (13, 53). Unlike benzodiazepines, which primarily accelerate GABA receptor activity (54), acupuncture promotes GABA synthesis and contributes to neural stabilization (53). These mechanisms may play a role in stabilization of mood and sleep-wake rhythms through neurotransmitter regulation (55), which may provide a potential explanation for the improvements in sleep anxiety and continuity observed in this review. Therefore, given its ability to reduce nighttime arousal and sleep anxiety without the risk of tolerance or dependence, acupuncture-related treatment is a promising complementary therapy for pediatric sleep disturbances, particularly in population with limited pharmacological options.

### 4.5 Strengths and limitations

This is the first meta-analysis evaluating acupuncture-related treatments as adjunctive therapies for sleep disturbances in children with NDDs. Overall, the findings suggest that these interventions offer potential benefits when combined with CTs. However, substantial heterogeneity resulting from differences in acupuncture modalities and non-pharmacological treatments across the studies requires careful interpretation of the results. None of the included studies mentioned blinding procedures, and only one study used sham acupuncture, eliminating the risk of expectancy and placebo effects associated with invasive procedures. Additionally, the uncertainty regarding the protocol registration of the studies, except for one (39), further raised concerns regarding selective reporting bias and limited methodological transparency. Despite these limitations, the CSHQ total score and TER were significantly higher in the acupuncture-related group, which is supported by moderate-quality evidence. Furthermore, improvements were observed in most sleep-related outcomes, indicating that acupuncture-related treatment is a promising non-pharmacological option for this population. Nevertheless, given the high risk of bias and limited methodological quality across the studies, these findings should be interpreted with caution. Moreover, as most studies were conducted in China, where acupuncture is culturally accepted, the results may be influenced by cultural and geographic bias, thereby limiting their generalizability.

### 4.6 Recommendations for future research

Future research should enhance the quality of evidence by increasing the sample size, clearly reporting blinding procedures, and ensuring study protocol registration. Although the subgroup analyses based on different acupuncture-related modalities did not reveal statistically significant differences in the CSHQ total scores or TER, these findings may have been limited by the small number of included studies. High-quality RCTs involving a larger number from diverse regions are warranted to compare specific acupuncture modalities and explore which approaches may be more effective in managing sleep disturbances in children with NDDs.

## 5 Conclusions

This meta-analysis suggests that acupuncture-related treatments may have beneficial effects on sleep disturbances in children with NDDs, with few AEs reported. These treatments may serve as potential adjunctive therapies to existing CTs. However, the results should be interpreted with caution due to methodological limitations across the included studies, including high risk of bias.

## Data Availability

The original contributions presented in the study are included in the article/**Supplementary Material**. Further inquiries can be directed to the corresponding author.

## Supplementary material

Supplementary Table 1 Search strategies for each database.

Supplementary Table 2 Evaluation of acupuncture-related treatments in the included studies using STRICTA criteria.

Supplementary Table 3 Characteristics of the included studies.

Supplementary Table 4 Detailed information on acupuncture-related treatment groups.

Supplementary Figure 1 Forest plot comparing acupuncture-related treatment plus CT vs. CT on CSHQ 8-subscale scores. AT, acupuncture; CT, conventional treatment; CSHQ, Children’s Sleep Habits Questionnaire

Supplementary Figure 2 Forest plot comparing acupuncture-related treatment plus CT vs. CT on CSHQ 5-subscale scores. AT, acupuncture; CT, conventional treatment; CSHQ, Children’s Sleep Habits Questionnaire

Supplementary Figure 3 Funnel plot showing publication bias in the CSHQ total score. ●: studies including the outcome. CSHQ, Children’s Sleep Habits Questionnaire

Supplementary Figure 4 Sensitivity analysis plot. **(A)** CSHQ total score; **(B)** Sleep anxiety (8-subscale CSHQ); **(C)** Sleep duration (8-subscale CSHQ); **(D)** Nighttime awakenings (8-subscale CSHQ); **(E)** Sleep-disordered breathing (8-subscale CSHQ); **(F)** Daytime sleepiness (8-subscale CSHQ); **(G)** Bedtime habits (5-subscale CSHQ); **(H)** Morning waking habits (5-subscale CSHQ); **(I)** Sleep behavior (5-subscale CSHQ); **(J)** Daytime sleepiness (5-subscale CSHQ); **(K)** TER; **(L)** Number of nighttime awakenings (PSG); **(M)** Sleep efficiency (PSG); **(N)** Nighttime sleep time (PSG). CSHQ, Children’s Sleep Habits Questionnaire; TER, total effective rate; PSG, polysomnography

## References

[1] Al Lihabi A . A literature review of sleep problems and neurodevelopment disorders. Front Psychiatry. (2023) 14:1122344. doi: 10.3389/fpsyt.2023.1122344, PMID: 36911135 PMC9995546

[2] Dutt R Roduta-Roberts M Brown CA . Sleep and children with cerebral palsy: A review of current evidence and environmental non-pharmacological interventions. Children (Basel). (2015) 2:78–88. doi: 10.3390/children2010078, PMID: 27417351 PMC4928749

[3] Kang YQ Song XR Wang GF Su YY Li PY Zhang X . Sleep problems influence emotional/behavioral symptoms and repetitive behavior in preschool-aged children with autism spectrum disorder in the unique social context of China. Front Psychiatry. (2020) 11:273. doi: 10.3389/fpsyt.2020.00273, PMID: 32372982 PMC7179767

[4] Sanabra M Gómez-Hinojosa T Grau N Alda JA . Deficient emotional self-regulation and sleep problems in adhd with and without pharmacological treatment. J Atten Disord. (2022) 26:426–33. doi: 10.1177/1087054720986242, PMID: 33472511

[5] Hosokawa R Tomozawa R Fujimoto M Anzai S Sato M Tazoe H . Association between sleep habits and behavioral problems in early adolescence: A descriptive study. BMC Psychol. (2022) 10:254. doi: 10.1186/s40359-022-00958-7, PMID: 36335370 PMC9636702

[6] Favole I Davico C Marcotulli D Sodero R Svevi B Amianto F . Sleep disturbances and emotional dysregulation in young children with autism spectrum, intellectual disability, or global developmental delay. Sleep Med. (2023) 105:45–52. doi: 10.1016/j.sleep.2023.02.026, PMID: 36963320

[7] Chen J Li H Zhong D Xu F Ding L Tang C . A bibliometric analysis of acupuncture for neurodevelopmental disorders: A call for increased output and future research priorities. Heliyon. (2023) 9:e22799. doi: 10.1016/j.heliyon.2023.e22799, PMID: 38213582 PMC10782164

[8] Bartels EM Korbo L Harrison AP . Novel insights into cerebral palsy. J Muscle Res Cell Motil. (2020) 41:265–7. doi: 10.1007/s10974-020-09577-4, PMID: 32065339

[9] Francés L Quintero J Fernández A Ruiz A Caules J Fillon G . Current state of knowledge on the prevalence of neurodevelopmental disorders in childhood according to the dsm-5: A systematic review in accordance with the prisma criteria. Child Adolesc Psychiatry Ment Health. (2022) 16:27. doi: 10.1186/s13034-022-00462-1, PMID: 35361232 PMC8973738

[10] Bruni O Angriman M Melegari MG Ferri R . Pharmacotherapeutic management of sleep disorders in children with neurodevelopmental disorders. Expert Opin Pharmacother. (2019) 20:2257–71. doi: 10.1080/14656566.2019.1674283, PMID: 31638842

[11] Hamilton A Joyce A Spiller J . Recommendations for assessing and managing sleep problems in children with neurodevelopmental conditions. Curr Dev Disord Rep. (2023) 10:274–85. doi: 10.1007/s40474-023-00283-7

[12] Cohen EM Dossett ML Mehta DH Davis RB Lee YC . Factors associated with insomnia and complementary medicine use in children: results of a national survey. Sleep Med. (2018) 44:82–8. doi: 10.1016/j.sleep.2018.01.007, PMID: 29530374 PMC5999317

[13] Yao L Liu Y Li M Zheng H Sun M He M . The central regulatory effects of acupuncture in treating primary insomnia: A review. Front Neurol. (2024) 15:1406485. doi: 10.3389/fneur.2024.1406485, PMID: 39719980 PMC11666528

[14] Lee B Lee J Cheon JH Sung HK Cho SH Chang GT . The efficacy and safety of acupuncture for the treatment of children with autism spectrum disorder: A systematic review and meta-analysis. Evid Based Complement Alternat Med. (2018) 2018:1057539. doi: 10.1155/2018/1057539, PMID: 29552077 PMC5820575

[15] Lai S Wan H Deng F Li Y An Y Peng J . Efficacy and safety of acupuncture for tourette syndrome in children: A meta-analysis and systematic review. Clin Pediat. (2024) 64:719–35. doi: 10.1177/00099228241283279, PMID: 39345099

[16] Ang L Kim JT Kim K Lee HW Choi JY Kim E . Acupuncture for treating attention deficit hyperactivity disorder in children: A systematic review and meta-analysis. Med (Kaunas). (2023) 59:392. doi: 10.3390/medicina59020392, PMID: 36837594 PMC9965965

[17] Lu C Wu LQ Hao H Kimberly Leow X Xu FW Li PP . Clinical efficacy and safety of acupuncture treatment of tic disorder in children: A systematic review and meta-analysis of 22 randomized controlled trials. Complement Ther Med. (2021) 59:102734. doi: 10.1016/j.ctim.2021.102734, PMID: 33989798

[18] Owens JA Spirito A McGuinn M . The children’s sleep habits questionnaire (Cshq): psychometric properties of a survey instrument for school-aged children. Sleep. (2000) 23:1043–51. doi: 10.1093/sleep/23.8.1d 11145319

[19] National H . Family planning commission of the people’s republic of C. In: Guideline for Sleep Hygiene among Children Aged 0～5 Years. China National Health and Family Planning Commission, Beijing, China (2017).

[20] MacPherson H Altman DG Hammerschlag R Youping L Taixiang W White A . Revised standards for reporting interventions in clinical trials of acupuncture (Stricta): extending the consort statement. PLoS Med. (2010) 7:e1000261. doi: 10.1371/journal.pmed.1000261, PMID: 20543992 PMC2882429

[21] Sterne JAC Savović J Page MJ Elbers RG Blencowe NS Boutron I . Rob 2: A revised tool for assessing risk of bias in randomised trials. BMJ. (2019) 366:l4898. doi: 10.1136/bmj.l4898, PMID: 31462531

[22] Egger M Davey Smith G Schneider M Minder C . Bias in meta-analysis detected by a simple, graphical test. BMJ. (1997) 315:629–34. doi: 10.1136/bmj.315.7109.629, PMID: 9310563 PMC2127453

[23] Guyatt GH Oxman AD Kunz R Vist GE Falck-Ytter Y Schünemann HJ . What is “Quality of evidence” and why is it important to clinicians? BMJ. (2008) 336:995–8. doi: 10.1136/bmj.39490.551019.BE, PMID: 18456631 PMC2364804

[24] Yu Z Kong M Zhang Y . Analysis of the efficacy of transcranial magnetic stimulation combined with scalp acupuncture in the treatment of cerebral palsy combined with sleep disorders. Chin J Integr Tradit West Med Pediatr. (2024) 16:175–8. doi: 10.3969/j.issn.16743865.2024.02.016

[25] Ma Y Wu L Ren M Zhang X Ban H Cao M . Moxibustion of du meridian and fuyang acupoints combined with multimedia sensory integration training for the treatment of spastic cerebral palsy with sleep disorders. J Tradit Chin Med. (2023) 38:170–4. doi: 10.16368/j.issn.1674-8999.2023.01.029

[26] Hu Y Huang R Luo X Qin Z Wei X Liu J . Observation on the efficacy of acupuncture combined with auricular acupoint taping in the treatment of sleep disorders in patients with autism. Shanghai J Acupunct Moxibust. (2023) 42:1277–81. doi: 10.13460/j.issn.1005-0957.2023.12.1277

[27] Zhu X . Effect of rapid needling at yu-mu points on sleep disorders in children with autism spectrum disorders. Henan Tradit Chin Med. (2023) 43:603–6. doi: 10.16367/j.issn.1003-5028.2023.04.0124

[28] Huang C . Clinical observation on the therapeutic effect of auricular point pressing combined with acupuncture in the treatment of sleep disorders in children with cerebral palsy. World J Sleep Med. (2022) 9:90–1. doi: 10.3969/j.issn.2095-7130.2022.01.031

[29] Xu X Yu W Li R Hou F Chen D He J . A study on the effect of auricular acupuncture in treating sleep disorders in children with autism spectrum disorder. Reflexol Rehabil Med. (2022) 3:47–50.

[30] Sa R Xu R Hou Y Bao J . Clinical observation on Mongolian moxibustion in the treatment of sleep disorders in children with cerebral palsy. J Med Pharm Chin Minorities. (2022) 28:20–2. doi: 10.16041/j.cnki.cn15-1175.2022.07.001

[31] Zhang L Bao C Huang Y Wang Y Wang N Sun Y . Effects of sensory integration training combined with intestinal regulation and acupuncture on abc scores and sleep disorders in children with autism. Liaoning Zhong Yi Za Zhi. (2022) 49:167–70. doi: 10.13192/j.issn.1000-1719.2022.04.045

[32] Li Q Zhao Q Wang K Liu N . Effect of fang’s head acupuncture on sleep disorders in children with autism spectrum disorders. Beijing J Tradit Chin Med. (2022) 41:679–81. doi: 10.16025/j.1674-1307.2022.06.026

[33] Dang H . Clinical study on auricular acupoint pressing combined with acupuncture in the treatment of sleep disorders in children with cerebral palsy. J Med Theory Pract. (2022) 35:513–5. doi: 10.19381/j.issn.1001-7585.2022.03.068

[34] Huang L Hong Y Ge P Ou P Zhuang W Wang J . Clinical efficacy study of jinsan’s treatment of core symptoms and sleep disorders in children with mild to moderate autism. Lishizhen Med Mater Med Res. (2021) 32:2447–50. doi: 10.3969/j.issn.1008-0805.2021.10.35

[35] Li R Yu W Xu X Chen D . Effect of auricular acupuncture combined with comprehensive intervention on sleep disorders in children with autism spectrum disorders. J Clin Nurs Pract. (2021) 7:57–60.

[36] Yu W Xu X Li R Yang J Wang W . Effect of auricular acupuncture on sleep quality in children with autism spectrum disorder. Shenzhen J Integr Tradit Chin West Med. (2020) 30:45–7. doi: 10.16458/j.cnki.1007-0893.2020.21.020

[37] Zeng Y Huang R Deng L Liu F . Observation on the therapeutic effect of acupuncture on sleep disorders in children with mental retardation. Shanghai J Acupunct Moxibust. (2015) 34:41–3. doi: 10.13460/j.issn.1005-0957.2015.09.0836

[38] Chen S Fang J Wang Y Guo A Yang L Ma X . Clinical observation on the therapeutic effect of acupoint nerve therapy on sleep disorders in children with autism. Med Innov Chin. (2014) 11:124–7. doi: 10.3969/j.issn.1674-4985.2014.15.044

[39] Mohammadi L Tagharrobi Z Sharifi K Sooki Z Zare M Zare Joshaghani F . The effect of auriculotherapy on sleep quality in children with attention deficit hyperactivity disorder: A randomized clinical trial. BMC Pediatr. (2025) 25:48. doi: 10.1186/s12887-024-05371-0, PMID: 39833716 PMC11744890

[40] Liu S Zhu M Li Y . Effects of jin’s three-needles therapy combined with low-frequency repetitive transcranial magnetic stimulation on sleep disorders and eeg in children with spastic cerebral palsy. Zhongguo zhen jiu = Chin acupuncture moxibustion. (2024) 44:1267–72. doi: 10.13703/j.0255-2930.20231219-k0004, PMID: 39532443

[41] Moher D Liberati A Tetzlaff J Altman DG . Preferred reporting items for systematic reviews and meta-analyses: the prisma statement. PLoS Med. (2009) 6:e1000097. doi: 10.1371/journal.pmed.1000097, PMID: 19621072 PMC2707599

[42] Markovich AN Gendron MA Corkum PV . Validating the children’s sleep habits questionnaire against polysomnography and actigraphy in school-aged children. Front Psychiatry. (2014) 5:188. doi: 10.3389/fpsyt.2014.00188, PMID: 25610402 PMC4285019

[43] Li J Ran X Cui C Xiang C Zhang A Shen F . Instant sedative effect of acupuncture at gv20 on the frequency of electroencephalogram Α and Β Waves in a model of sleep deprivation. Exp Ther Med. (2018) 15:5353–8. doi: 10.3892/etm.2018.6123, PMID: 29896222 PMC5994783

[44] Lu G Chen F Guo C Wu J . Acupuncture for senile insomnia: A systematic review of acupuncture point. Arch Gerontology Geriatrics. (2024) 127:105586. doi: 10.1016/j.archger.2024.105586, PMID: 39096556

[45] Park HJ Park HJ Chae Y Kim JW Lee H Chung J-H . Effect of acupuncture on hypothalamic-pituitary-adrenal system in maternal separation rats. Cell Molec Neurobiol. (2011) 31:1123–7. doi: 10.1007/s10571-011-9718-x, PMID: 21643998 PMC11498392

[46] Seo SY Ryu Y . Electroacupuncture stimulation of ht7 alleviates sleep disruption following acute caffeine exposure by regulating bdnf-mediated endoplasmic reticulum stress in the rat medial septum. BioMed Pharmacother. (2022) 155:113724. doi: 10.1016/j.biopha.2022.113724, PMID: 36156370

[47] Luo WZ Zhang QZ Lai XS . Effect of acupuncture treatment of relieving depression and regulating mind on insomnia accompanied with depressive disorders. Zhongguo Zhen Jiu. (2010) 30:899–903. doi: 10.13703/j.0255-2930.2010.11.005, PMID: 21246844

[48] Li YW Li W Wang ST Gong YN Dou BM Lyu ZX . The autonomic nervous system: A potential link to the efficacy of acupuncture. Front Neurosci. (2022) 16:1038945. doi: 10.3389/fnins.2022.1038945, PMID: 36570846 PMC9772996

[49] Robinson-Shelton A Malow BA . Sleep disturbances in neurodevelopmental disorders. Curr Psychiatry Rep. (2015) 18:6. doi: 10.1007/s11920-015-0638-1, PMID: 26719309

[50] Beopoulos A Gea M Fasano A Iris F . Autonomic nervous system neuroanatomical alterations could provoke and maintain gastrointestinal dysbiosis in autism spectrum disorder (Asd): A novel microbiome-host interaction mechanistic hypothesis. Nutrients. (2021) 14:65. doi: 10.3390/nu14010065, PMID: 35010940 PMC8746684

[51] Li QQ Shi GX Xu Q Wang J Liu CZ Wang LP . Acupuncture effect and central autonomic regulation. Evid Based Complement Alternat Med. (2013) 2013:267959. doi: 10.1155/2013/267959, PMID: 23762116 PMC3677642

[52] Zhu W Huang L Cheng H Li N Zhang B Dai W . Gaba and its receptors’ Mechanisms in the treatment of insomnia. Heliyon. (2024) 10:e40665. doi: 10.1016/j.heliyon.2024.e40665, PMID: 39654705 PMC11626785

[53] Lee S Kim S-N . The effects of acupuncture on sleep disorders and its underlying mechanism: A literature review of rodent studies. Front Neurosci. (2023) 17:1243029. doi: 10.3389/fnins.2023.1243029, PMID: 37614343 PMC10442542

[54] Goldschen-Ohm MP . Benzodiazepine modulation of gaba(a) receptors: A mechanistic perspective. Biomolecules. (2022) 12:1784. doi: 10.3390/biom12121784, PMID: 36551212 PMC9775625

[55] Landolt HP Holst SC Valomon A . Clinical and experimental human sleep-wake pharmacogenetics. Handb Exp Pharmacol. (2019) 253:207–41. doi: 10.1007/164_2018_175, PMID: 30443785

