# Supplementary material for: Efficacy of acupuncture-related treatment for sleep disturbances in children with neurodevelopmental disorders: a systematic review and meta-analysis
Source: Front Psychiatry. 2026 Jan 13;16:1670438. doi: 10.3389/fpsyt.2025.1670438 (PMC12835266; doi:10.3389/fpsyt.2025.1670438)
Supplement: Supplementary Table 1 — Search strategies for each database. [file Table1.docx]

Supplemental Material

# Supplementary Table 1. Search strategies for each database

**CNKI**

|  | Searches | Results |
| --- | --- | --- |
| #1 | (SU=’神经发育*’+’脑瘫’+’脑性瘫痪’+'Cerebral palsy'+’注意力缺陷多动障碍*’+’注意力缺陷’+’多动症’+’多动*’+’孤独症谱系障碍’+’孤独症’+’自闭症谱系障碍’+’自闭症’+’图雷特综合征’+’抽动障碍*’+’抽动样’+’抽动综合征’+’智力残疾*’+’智力低下’+’智力障碍*’+’沟通障碍*’+’言语或语言障碍*’+’言语障碍’+’语言障碍’+‘学习障碍*’+’阅读障碍’+’计算障碍’+’书写障碍’+’协调障碍*’+’刻板运动障碍*’+‘发育性协调障碍’) AND (SU='失眠*'+‘睡眠不安’+’睡眠焦虑’+’睡眠障碍’+’睡眠紊乱‘+’睡眠觉醒障碍‘+’睡眠启动与维持障碍’+'insomnia'+’sleep restlessness’+'sleep disorder'+'sleep disturbance') AND (SU='小儿'+'儿童'+'儿'+'小孩'+'孩子'+’青少年’) AND (SU='针'+'刺法'+'灸'+'埋线'+'蜂毒'+'蜂疗'+'药物穿刺'+'穴位'+'腧穴'+'指压'+'放血'+'刺络’) AND (SU='随机'+'对照'+'随意'+'试验'+'安慰') | **31** |

**Wangfang**

|  | Searches | Results |
| --- | --- | --- |
| #1 | TI=(神经发育* OR 脑瘫 OR 脑性瘫痪 OR Cerebral palsy OR 注意力缺陷多动障碍* OR 注意力缺陷 OR 多动症 OR 多动* OR 孤独症谱系障碍 OR 孤独症 OR 自闭症谱系障碍 OR 自闭症 OR 图雷特综合征 OR 抽动障碍* OR 抽动样 OR 抽动综合征 OR 智力残疾* OR 智力低下 OR 智力障碍* OR 沟通障碍* OR 言语或语言障碍* OR 言语障碍 OR 语言障碍 OR 学习障碍* OR 阅读障碍 OR 计算障碍 OR 书写障碍 OR 协调障碍* OR 刻板运动障碍* OR 发育性协调障碍) AND TI=(失眠* OR 睡眠不安 OR 睡眠焦虑 OR 睡眠障碍 OR 睡眠紊乱 OR 睡眠觉醒障碍 OR 睡眠启动与维持障碍 OR insomnia OR sleep restlessness OR sleep disorder OR sleep disturbance) AND AB=(小儿 OR 儿童 OR 儿 OR 小孩 OR 孩子 OR 青少年) AND TI=(针 OR 电针 OR 刺法 OR 灸 OR 埋线 OR 蜂毒 OR 蜂疗 OR 药物穿刺 OR 穴位 OR 腧穴 OR 指压 OR 放血 OR 刺络) AND AB=(随机 OR 对照 OR 随意 OR 试验 OR 安慰) | **28** |

**VIP**

|  | Searches | Results |
| --- | --- | --- |
| #1 | M=(神经发育 OR 脑瘫 OR 脑性瘫痪 OR Cerebral palsy OR 注意力缺陷多动障碍 OR 注意力缺陷 OR 多动症 OR 多动 OR 孤独症谱系障碍 OR 孤独症 OR 自闭症谱系障碍 OR 自闭症 OR 图雷特综合征 OR 抽动障碍 OR 抽动样 OR 抽动综合征 OR 智力残疾 OR 智力低下 OR 智力障碍 OR 沟通障碍 OR 言语或语言障碍 OR 言语障碍 OR 语言障碍 OR 学习障碍 OR 阅读障碍 OR 计算障碍 OR 书写障碍 OR 协调障碍 OR 刻板运动障碍 OR 发育性协调障碍) AND M=(失眠 OR 睡眠不安 OR 睡眠焦虑 OR 睡眠障碍 OR 睡眠紊乱 OR 睡眠觉醒障碍 OR 睡眠启动与维持障碍 OR insomnia OR sleep restlessness OR sleep disorder OR sleep disturbance) AND M=(小儿 OR 儿童 OR 儿 OR 小孩 OR 孩子 OR 青少年) AND M=(针 OR 电针 OR 刺法 OR 灸 OR 埋线 OR 蜂毒 OR 蜂疗 OR 药物穿刺 OR 穴位 OR 腧穴 OR 指压 OR 放血 OR 刺络) AND M=(随机 OR 对照 OR 随意 OR 试验 OR 安慰) | **10** |

**Pubmed**

|  | Searches | Results |
| --- | --- | --- |
| #1 | “neurodevelopmental*” OR “cerebral palsy” OR “attention deficit hyperactivity disorder*” OR “attention deficit” OR “hyperactivity*” OR “autism spectrum disorder” OR “autism*” OR “autistic disorder” OR “Tourette syndrome” OR “Tourette*” OR “tic disorder*” OR “tic-like” OR “tic-related” OR “tic syndrome*” OR “intellectual disabilit*” OR “mental retardation” OR “intellectual disability” OR “communication disorder*” OR “speech or language disorder*” OR “speech disorder” OR “speech sound disorder” OR “language disorder” OR “learning disorder*” OR ”learning disabilt*“ OR ”learning-dis*“ OR ”reading disorder“ OR ”dyscalculia“ OR ”dysgraphia“ OR “coordination disorder*” OR “stereotyped movement disorder*” OR “developmental coordination disorder” | 346,439 |
| #2 | “Insomnia” OR “sleep restlessness” OR “sleep disturbance” OR “Sleep Wake Disorders”[MeSH Terms] OR “Sleep Initiation and Maintenance Disorders”[MeSH Terms] | 142,333 |
| #3 | “Child” [MeSH Terms] OR “Pediatrics” [MeSH Terms] OR “Adolescent” [MeSH Terms] | 3,560,195 |
| #4 | “acupuncture”[MeSH Terms] OR “acupuncture Therapy”[MeSH Terms] OR “acupressure”[MeSH Terms] OR　“Electroacupuncture”[MeSH Terms] OR “auriculotherapy”[MeSH Terms] OR “acupuncture points”[MeSH Terms] OR “acupuncture, ear”[MeSH Terms] OR “acupunct*”[TIAB] OR “needl*”[TIAB] OR “acupoint*”[TIAB] OR “electroacupuncture*”[TIAB] OR “trigger point*”[TIAB] OR “moxa”[TIAB] OR “pharmacopuncture*”[TIAB] OR “catgut embedding”[TIAB] OR “catgut implantation”[TIAB] OR “acupotom*”[TIAB] OR “shiatsu”[TIAB] OR “Zhi Ya”[TIAB] OR “bee venom*”[TIAB] OR “bee sting therapy”[TIAB] OR “apitoxin”[TIAB] OR “apitherap*”[TIAB] OR “phlebotom*”[TIAB] OR “flebotom*”[TIAB] OR “venesection*”[TIAB] OR “venipuncture*”[TIAB] OR “bloodletting”[TIAB] OR “blood letting”[TIAB] | 209,652 |
| #5 | “randomized controlled trial”[PT] OR “controlled clinical trial”[PT] OR “randomized”[TIAB] OR “randomly”[TIAB] OR “trial”[TIAB] | 1,805,256 |
| #6 | “animals” [MeSH Terms] NOT “humans” [MeSH Terms] | 5,332,362 |
| #7 | #1 AND #2 AND #3 AND #4 AND #5 NOT #6 | **7** |

**Embase via Elsevier**

|  | Searches | Results |
| --- | --- | --- |
| #1 | ‘neurodevelopmental*’/exp OR ‘cerebral palsy’/exp OR 'attention deficit hyperactivity disorder*'/exp OR ‘attention deficit’/exp OR hyperactivity*/exp OR ‘autism spectrum disorder’/exp OR autism*/exp OR ‘autistic disorder’/exp OR ‘Tourette syndrome’/exp OR ‘Tourette*’/exp OR ‘tic disorder*’/exp OR ‘tic-like’/exp OR ‘tic-related’/exp OR ‘tic syndrome*’/exp OR ‘intellectual disabilit*’/exp OR ‘mental retardation’/exp OR ‘intellectual disability’/exp OR ‘communication disorder*’/exp OR ‘speech or language disorder*’/exp OR ‘speech disorder’/exp OR ‘speech sound disorder’/exp OR ‘language disorder’/exp OR ‘learning disorder*’/exp OR ‘learning disabilt*’/exp OR ‘learning-dis*’/exp OR ‘reading disorder’/exp OR ‘dyscalculia’/exp OR ‘dysgraphia’/exp OR ‘coordination disorder*’/exp OR ‘stereotyped movement disorder*’/exp OR ‘developmental coordination disorder’/exp | 1,207,731 |
| #2 | Insomnia/exp OR ‘sleep restlessness’/exp OR ‘sleep disturbance’/exp OR ‘Sleep Wake Disorders’/exp OR ‘Sleep Initiation and Maintenance Disorders’/exp | 380,642 |
| #3 | child/exp OR pediatrics/exp OR infant/exp OR adolescent/exp OR child*:ab,ti OR pediatric*:ab,ti OR infant:ab,ti OR adolescent:ab,ti | 5,406,118 |
| #4 | acupuncture/exp OR acupressure/exp OR 'acupuncture needle'/exp OR 'acupuncture point'/exp OR electroacupuncture/exp OR ‘auricular acupuncture’/exp OR 'trigger point'/exp OR moxibustion/exp OR 'bee venom'/exp OR apitherapy/exp OR phlebotomy/exp OR bloodletting/exp OR acupunct*:ab,ti OR needl*:ab,ti OR acupoint*:ab,ti OR moxa:ab,ti OR pharmacopuncture*:ab,ti OR 'catgut embedding':ab,ti OR 'catgut implantation':ab,ti OR acupotom*:ab,ti OR shiatsu:ab,ti OR shiatzu:ab,ti OR 'Zhi Ya':ab,ti OR 'Chih Ya':ab,ti OR 'bee venom*':ab,ti OR 'bee sting therapy':ab,ti OR apitoxin:ab,ti OR apitherap*:ab,ti OR phlebotom*:ab,ti OR flebotom*:ab,ti OR venesection*:ab,ti OR venipuncture*:ab,ti OR bloodletting:ab,ti OR 'blood letting':ab,ti | 322,226 |
| #5 | 'crossover procedure':de OR 'double-blind procedure':de OR 'randomized controlled trial':de OR 'single-blind procedure':de OR (random* OR factorial* OR crossover* OR cross NEXT/1 over* OR doubl* NEAR/1 blind* OR singl* NEAR/1 blind* OR assign* OR allocat* OR volunteer*):de,ab,ti | 3,345,647 |
| #6 | #1 AND #2 AND #3 AND #4 AND #5 | 30 |
| #7 | #6 AND 'randomized controlled trial'/de | **9** |

**Central**

|  | Searches | Results |
| --- | --- | --- |
| #1 | MeSH descriptor: [Sleep Wake Disorders] explode all trees | 12,601 |
| #2 | MeSH descriptor: [Sleep Initiation and Maintenance Disorders] explode all trees | 3,939 |
| #3 | MeSH descriptor: [Sleep Deprivation] explode all trees | 1,143 |
| #4 | #1 OR #2 OR #3 | 12,601 |
| #5 | (neurodevelopmental* OR cerebral palsy OR attention deficit hyperactivity disorder* OR attention deficit OR hyperactivity* OR autism spectrum disorder OR autism* OR autistic disorder OR Tourette syndrome OR Tourette* OR tic disorder* OR tic-like OR tic-related OR tic syndrome* OR intellectual disabilit* OR mental retardation OR communication disorder* OR speech or language disorder* OR speech disorder OR speech sound disorder OR language disorder OR learning disorder* OR learning disabilt* OR learning-dis* OR reading disorder OR dyscalculia OR dysgraphia OR coordination disorder* OR stereotyped movement disorder* OR developmental coordination disorder):ti,ab,kw | 52,183 |
| #6 | #4 AND #5 | 588 |
| #7 | MeSH descriptor: [Child] explode all trees | 81,919 |
| #8 | MeSH descriptor: [Pediatrics] explode all trees | 1,052 |
| #9 | MeSH descriptor: [Infant] explode all trees | 45,528 |
| #10 | MeSH descriptor: [Adolescent] explode all trees | 136,477 |
| #11 | MeSH descriptor: [Minors] explode all trees | 13 |
| #12 | #7 OR #8 OR #9 OR #10 OR #11 | 204,173 |
| #13 | MeSH descriptor: [Acupuncture] explode all trees | 216 |
| #14 | MeSH descriptor: [Acupuncture Points] explode all trees | 2,825 |
| #15 | MeSH descriptor: [Acupuncture Therapy] explode all trees | 7,123 |
| #16 | MeSH descriptor: [Electroacupuncture] explode all trees | 1,172 |
| #17 | MeSH descriptor: [Acupuncture, Ear] explode all trees | 271 |
| #18 | MeSH descriptor: [Acupressure] explode all trees | 606 |
| #19 | MeSH descriptor: [Auriculotherapy] explode all trees | 328 |
| #20 | MeSH descriptor: [Moxibustion] explode all trees | 688 |
| #21 | MeSH descriptor: [Bee Venoms] explode all trees | 57 |
| #22 | MeSH descriptor: [Apitherapy] explode all trees | 31 |
| #23 | MeSH descriptor: [Phlebotomy] explode all trees | 622 |
| #24 | (acupunct* OR needl* OR acupoint* OR electroacupuncture* OR trigger point* OR acupressure OR moxibustion OR moxa OR pharmacopuncture* OR catgut embedding OR catgut implantation OR acupotom* OR shiatsu OR shiatzu OR Zhi Ya OR Chih Ya OR bee venom* OR bee sting therapy OR apitoxin OR apitherap* OR phlebotom* OR flebotom* OR venesection* OR venipuncture* OR bloodletting OR blood letting):ti,ab,kw | 52,568 |
| #25 | #13 OR #14 OR #15 OR #16 OR #17 OR #18 OR #19 OR #20 OR #21 OR #22 OR #23 OR #24 | 52,605 |
| #26 | (#6 AND #12 AND #25) in Trials | **7** |

**Web of science**

|  | Searches | Results |
| --- | --- | --- |
| #1 | TI=(“neurodevelopmental*” OR “cerebral palsy” OR “attention deficit hyperactivity disorder*” OR “attention deficit” OR “hyperactivity*” OR “autism spectrum disorder” OR “autism*” OR “autistic disorder” OR “Tourette syndrome” OR “Tourette*” OR “tic disorder*” OR “tic-like” OR “tic-related” OR “tic syndrome*” OR “intellectual disabilit*” OR “mental retardation” OR “communication disorder*” OR “speech or language disorder*” OR “speech disorder” OR “speech sound disorder” OR “language disorder” OR “learning disorder*” OR ”learning disabilt*“ OR ”learning-dis*“ OR ”reading disorder“ OR ”dyscalculia“ OR ”dysgraphia“ OR “coordination disorder*” OR “stereotyped movement disorder*” OR “developmental coordination disorder”) | 154,845 |
| #2 | TI=(“Insomnia” OR “sleep restlessness” OR “sleep disturbance” OR “Sleep Wake Disorders” OR “Sleep Initiation and Maintenance Disorders”) | 19,589 |
| #3 | AB=(“Child*” OR “Pediatric*” OR “Adolescent*”) | 1,729,828 |
| #4 | AB=(“Acupuncture*” OR “Acupressure” OR “acupoint*” OR “Electroacupuncture*” OR “Auriculotherapy” OR “Moxibustion” OR “Bee Venom*” OR “Apitherapy” OR “Phlebotomy” OR “needl*” OR “trigger point*” OR “pharmacopuncture*” OR “catgut embedding” OR “bloodletting” OR “blood letting”) | 176,372 |
| #5 | AB=(“randomized controlled trial” OR “controlled clinical trial” OR ”randomized” OR ”randomly” OR ”trial”) | 1,615,564 |
| #6 | #1 AND #2 AND #3 AND #4 AND #5 | **0** |

**SCOPUS**

|  | Searches | Results |
| --- | --- | --- |
| #1 | TITLE-ABS(“neurodevelopmental*” OR “cerebral palsy” OR “attention deficit hyperactivity disorder*” OR “attention deficit” OR “hyperactivity*” OR “autism spectrum disorder” OR “autism*” OR “autistic disorder” OR “Tourette syndrome” OR “Tourette*” OR “tic disorder*” OR “tic-like” OR “tic-related” OR “tic syndrome*” OR “intellectual disabilit*” OR “mental retardation” OR “communication disorder*” OR “speech or language disorder*” OR “speech disorder” OR “speech sound disorder” OR “language disorder” OR “learning disorder*” OR ”learning disabilit*“ OR ”learning-dis*“ OR ”reading disorder“ OR ”dyscalculia“ OR ”dysgraphia“ OR “coordination disorder*” OR “stereotyped movement disorder*” OR “developmental coordination disorder”) AND TITLE-ABS(“Insomnia” OR “sleep restlessness” OR “sleep disturbance” OR “Sleep Wake Disorders” OR “Sleep Initiation and Maintenance Disorders”) AND TITLE-ABS(“Child*” OR “Pediatric*” OR “Adolescent*”) AND TITLE-ABS(“Acupuncture*” OR “Acupressure” OR “acupoint*” OR “Electroacupuncture*” OR “Auriculotherapy” OR “Moxibustion” OR “Bee Venom*” OR “Apitherapy” OR “Phlebotomy” OR “needl*” OR “trigger point*” OR “pharmacopuncture*” OR “catgut embedding” OR “bloodletting” OR “blood letting”) AND TITLE-ABS(“randomized controlled trial” OR “controlled clinical trial” OR ”randomized” OR ”randomly” OR ”trial”) | **5** |

**AMED via EBSCO**

|  | Searches | Results |
| --- | --- | --- |
| S6 | S1 AND S2 AND S3 AND S4 AND S5 | **0** |
| S5 | TX (randomized controlled trial OR controlled clinical trial OR randomized crossover procedure OR double-blind procedure OR randomized controlled trial OR single-blind procedure OR random* OR factorial* OR crossover* OR double blind* OR assign* OR allocat*) | 30,380 |
| S4 | SU (Acupuncture* OR Acupuncture Therapy OR Acupressure OR Electroacupuncture OR Auriculotherapy OR Moxibustion OR Bee Venoms OR Apitherapy OR Phlebotomy OR acupunct* OR needl* OR acupoint* OR electroacupuncture* OR trigger point* OR moxa OR pharmacopuncture* OR catgut embedding OR catgut implantation OR acupotom* OR shiatsu OR shiatzu OR Zhi Ya OR Chih Ya OR bee venom* OR bee sting therapy OR apitoxin OR apitherap* OR phlebotom* OR flebotom* OR venesection* OR venipuncture* OR bloodletting OR blood letting) | 12,451 |
| S3 | SU (Child* OR Pediatric* OR Infant* OR Adolescent* OR Minors) | 27,008 |
| S2 | SU (Insomnia OR sleep restlessness OR sleep disturbance* OR Sleep Wake Disorder* OR Sleep Initiation and Maintenance Disorder*) | 425 |
| S1 | SU (neurodevelopmental* OR cerebral palsy OR attention deficit hyperactivity disorder* OR attention deficit OR hyperactivity* OR autism spectrum disorder OR autism* OR autistic disorder OR Tourette syndrome OR Tourette* OR tic disorder* OR tic-like OR tic-related OR tic syndrome* OR intellectual disabilit* OR mental retardation OR communication disorder* OR speech or language disorder* OR speech disorder OR speech sound disorder OR language disorder OR learning disorder* OR learning disabilt* OR learning-dis* OR reading disorder OR dyscalculia OR dysgraphia OR coordination disorder* OR stereotyped movement disorder* OR developmental coordination disorder) | 16,006 |

**MEDLINE via EBSCO**

|  | Searches | Results |
| --- | --- | --- |
| S6 | S1 AND S2 AND S3 AND S4 AND S5 | **4** |
| S5 | PT (randomized controlled trial OR controlled clinical trial OR randomized crossover procedure OR double-blind procedure OR randomized controlled trial OR single-blind procedure OR random* OR factorial* OR crossover* OR double blind* OR assign* OR allocat*) | 732,469 |
| S4 | SU (Acupuncture* OR Acupuncture Therapy OR Acupressure OR Electroacupuncture OR Auriculotherapy OR Moxibustion OR Bee Venoms OR Apitherapy OR Phlebotomy OR acupunct* OR needl* OR acupoint* OR electroacupuncture* OR trigger point* OR moxa OR pharmacopuncture* OR catgut embedding OR catgut implantation OR acupotom* OR shiatsu OR shiatzu OR Zhi Ya OR Chih Ya OR bee venom* OR bee sting therapy OR apitoxin OR apitherap* OR phlebotom* OR flebotom* OR venesection* OR venipuncture* OR bloodletting OR blood letting) | 153,323 |
| S3 | SU (Child* OR Pediatric* OR Infant* OR Adolescent* OR Minors) | 4,361,266 |
| S2 | SU (Insomnia OR sleep restlessness OR sleep disturbance OR Sleep Wake Disorders OR Sleep Initiation and Maintenance Disorders) | 52,737 |
| S1 | SU (neurodevelopmental* OR cerebral palsy OR attention deficit hyperactivity disorder* OR attention deficit OR hyperactivity* OR autism spectrum disorder OR autism* OR autistic disorder OR Tourette syndrome OR Tourette* OR tic disorder* OR tic-like OR tic-related OR tic syndrome* OR intellectual disabilit* OR mental retardation OR communication disorder* OR speech or language disorder* OR speech disorder OR speech sound disorder OR language disorder OR learning disorder* OR learning disabilt* OR learning-dis* OR reading disorder OR dyscalculia OR dysgraphia OR coordination disorder* OR stereotyped movement disorder* OR developmental coordination disorder) | 319,917 |

**ICTRP**

|  | Searches (search for clinical trials in children) | Results |
| --- | --- | --- |
| #1 | (“neurodevelopmental*” OR “cerebral palsy” OR “attention deficit hyperactivity disorder*” OR “attention deficit” OR “hyperactivity*” OR “autism spectrum disorder” OR “autism*” OR “autistic disorder” OR “Tourette syndrome” OR “Tourette*” OR “tic disorder*” OR “tic-like” OR “tic-related” OR “tic syndrome*” OR “intellectual disabilit*” OR “mental retardation” OR “communication disorder*” OR “speech or language disorder*” OR “speech disorder” OR “speech sound disorder” OR “language disorder” OR “learning disorder*” OR ”learning disabilt*“ OR ”learning-dis*“ OR ”reading disorder“ OR ”dyscalculia“ OR ”dysgraphia“ OR “coordination disorder*” OR “stereotyped movement disorder*” OR “developmental coordination disorder”) AND (“Insomnia” OR “sleep restlessness” OR “sleep disturbance” OR “Sleep Wake Disorders” OR “Sleep Initiation and Maintenance Disorders”) AND (“Acupuncture*” OR “Acupressure” OR “acupoint*” OR “Electroacupuncture*” OR “Auriculotherapy” OR “Moxibustion” OR “Bee Venom*” OR “Apitherapy” OR “Phlebotomy” OR “needl*” OR “trigger point*” OR “pharmacopuncture*” OR “catgut embedding” OR “bloodletting” OR “blood letting”) | **0** |

**ClinicalTrials.gov**

|  | Searches | Results |
| --- | --- | --- |
| #1 | (“neurodevelopmental*” OR “cerebral palsy” OR “attention deficit hyperactivity disorder*” OR “attention deficit” OR “hyperactivity*” OR “autism spectrum disorder” OR “autism*” OR “autistic disorder” OR “Tourette syndrome” OR “Tourette*” OR “tic disorder*” OR “tic-like” OR “tic-related” OR “tic syndrome*” OR “intellectual disabilit*” OR “mental retardation” OR “communication disorder*” OR “speech or language disorder*” OR “speech disorder” OR “speech sound disorder” OR “language disorder” OR “learning disorder*” OR ”learning disabilt*“ OR ”learning-dis*“ OR ”reading disorder“ OR ”dyscalculia“ OR ”dysgraphia“ OR “coordination disorder*” OR “stereotyped movement disorder*” OR “developmental coordination disorder”) AND (“Insomnia” OR “sleep restlessness” OR “sleep disturbance” OR “Sleep Wake Disorders” OR “Sleep Initiation and Maintenance Disorders”) AND (“Acupuncture*” OR “Acupressure” OR “acupoint*” OR “Electroacupuncture*” OR “Auriculotherapy” OR “Moxibustion” OR “Bee Venom*” OR “Apitherapy” OR “Phlebotomy” OR “needl*” OR “trigger point*” OR “pharmacopuncture*” OR “catgut embedding” OR “bloodletting” OR “blood letting”) | **0** |

**OASIS**

|  | Searches | Results |
| --- | --- | --- |
| #1 | (신경발달장애\|뇌성마비\|자폐\|ADHD\|) (소아\|아동\|청소년\|어린이) (침\|경혈\|혈위) | **1** |

**KMbase**

|  | Searches | Results |
| --- | --- | --- |
| #1 | Title(신경발달장애) OR Title(뇌성마비) OR Title(자폐) OR Title(ADHD) | 1,757 |
| #2 | Title(수면장애) OR Title(불면증) OR Title(수면) | 2,221 |
| #3 | ABSTRACT(소아) OR ABSTRACT(청소년) OR ABSTRACT(아동) OR ABSTRACT(어린이) | 32,708 |
| #4 | ABSTRACT(침) OR ABSTRACT(경혈) OR ABSTRACT(혈위) | 64,405 |
| #5 | #1 AND #2 AND #3 AND #4 | **0** |

**KISS**

|  | Searches | Results |
| --- | --- | --- |
| #1 | 초록=(신경발달장애\|뇌성마비\|자폐\|ADHD\|) AND 초록=(소아\|아동) AND 초록=(침\|경혈\|혈위) | **0** |

**CiNII**

|  | Searches | Results |
| --- | --- | --- |
| #1 | (神経発達* OR 神经发育障碍* OR 精神遅滞 OR 脳性麻痺 OR 注意欠陥多動性障害* OR 注意欠陥 OR 多動性* OR 孤独症谱系障碍 OR 自閉症* OR 自閉スペクトラム OR 自閉症スペクトラム OR トゥレット症候群 OR トゥレット* OR チック障害* OR チック様 OR チック関連 OR チック症候群* OR 知的障害* OR コミュニケーション障害* OR 言語障害* OR 言語音障害 OR 学習障害* OR 読字障害 OR 計算障害 OR 書字障害 OR 協調運動障害* OR　定型運動障害*　OR 発達性協調運動障害 OR 運動調整障害 OR 運動能力障害* OR 常同運動障害*) AND (不眠症 OR 睡眠不安 OR 睡眠不穏 OR 睡眠障害 OR 不眠 OR insomnia OR sleep restlessness OR sleep disorder OR sleep disturbance) AND (小児 OR 幼子 OR 児子 OR 子 OR 児 OR 児童 OR 幼児 OR 乳兒 OR 子供 OR 思春期の OR 青春の) AND (針 OR 鍼 OR 灸 OR 蜂毒 OR 蜂療法 OR 蜂針療法 OR 穴位注射 OR 穴 OR 壺 OR 圧痛点 OR 指圧 OR 瀉血 OR 刺絡) AND (ランダム化比較試験 OR 対照臨床試験 OR ランダム OR 無作為 OR 対照 OR 試験 OR 偽薬) | **0** |

**Supplementary Table 2. Evaluation of acupuncture-related treatments in the included studies using STRICTA criteria.**

| **No.** | **Study ID** | **Acupuncture rationale** | | | **Details of needling** | | | | | | | **Treatment regimen** | | **Other treatment components** | | **Practitioner background** | **Control intervention** |
| --- | --- | --- | --- | --- | --- | --- | --- | --- | --- | --- | --- | --- | --- | --- | --- | --- | --- |
|  |  | **Style of needle** | **Reasoning for treatment** | **Extent to which treatment was varied** | **No. of needles inserted** | **Points used** | **Depth of insertion** | **Response sought** | **Needle stimulation** | **Needle retention** | **Type of needle-related therapy** | **No. of treatment sessions** | **Frequency and duration** | **Other interventions** | **Setting and context of treatment** |  |  |
| **21** | Yu 2024 | Y | Y | NR | NR | Y^3,4^ | NR | Y | Y | Y | NR | Y | Y | Y | Y | NR | Y |
| **22** | Ma 2023 | Y | Y | NR | Y | Y | NA | NR | NA^1^ | Y | Y | Y | Y | Y | Y | NR | Y |
| **23** | Hu 2023 | Y | Y | Y^1^ | NR | Y^3,4^ | Y | NR | Y | Y | Y | Y | Y | Y | Y | NR | Y |
| **24** | Zhu 2023 | Y | Y | NR | NR | Y^3^ | Y^6^ | NR | Y | Y | Y | Y | Y | Y | Y | Y | Y |
| **25** | Huang 2022 | Y | Y | Y^1, 2^ | NR | Y^3,4^ | NR | Y | Y | Y | Y | Y | Y | Y | Y | NR | Y |
| **26** | Xu 2022 | Y | Y | NR | Y^3^ | Y^3^ | NR | NR | N^1^ | Y | Y | Y | Y | Y | Y | Y | Y |
| **27** | Sa 2022 | Y | Y | NR | NR | Y | NA | NR | NA^1^ | Y | Y | Y | Y | Y | Y | NR | Y |
| **28** | Zhang 2022 | Y | Y | NR | Y | Y | NR | NR | Y | Y | NR | Y | Y | Y | Y | NR | Y |
| **29** | Li 2022 | Y | Y | NR | NR | Y^3^ | NR | NR | Y | Y | Y | Y | Y | Y | Y | NR | Y |
| **30** | Dang 2022 | Y | Y | NR | NR | Y^3,4^ | NR | NR | Y^7^ | NR | Y | NR | NR^1^ | Y | Y | NR | Y |
| **31** | Huang 2021 | Y | Y | Y^1^ | Y | Y^4^ | Y | NR | Y | Y^8^ | Y | Y | Y | Y | Y | NR | Y |
| **32** | Li 2021 | Y | Y | NR | Y^4^ | Y^4^ | Y | NR | NR^2^ | Y | Y | Y | Y | Y | Y | NR | Y |
| **33** | Yu 2020 | Y | Y | NR | NR | Y^3^ | NR | NR | NR^2^ | Y | Y | Y | Y | Y | Y | NR | Y |
| **34** | Zeng 2015 | Y | Y | Y^2^ | NR | Y^4^ | Y | NR | Y | Y | Y | Y | Y | Y | Y | NR | Y |
| **35** | Chen 2014 | Y | Y | NR | Y | Y | NA | NR | Y | Y^9^ | Y | Y | Y | Y | Y | NR | Y |
| **36** | Mohammadi 2025 | Y | Y | NR | Y | Y | NR | NR | Y | Y | Y | Y | Y | Y | Y | Y | Y |
| **37** | Liu 2024 | Y | Y | NR | NR | Y^3^ | Y^5^ | Y | Y | Y | Y | Y | Y | Y | Y | Y | Y |

Notes: Y: Yes; Y^1^: Added acupoints by visceral pattern identification; Y^2^: Added acupoints by symptoms; Y^3^: Reported but did not mention unilateral or bilateral; Y^4^: Reported but points were not fixed; Y^5^: Mentioned but only described for certain acupuncture points;Y^6^: Mentioned but depended on the child’s fatness; Y^7^: Mentioned in acupressure, but not reported in acupuncture; Y^8^: Mentioned in acupuncture, but no active stimulation in press needle; Y^9^: Electrical stimulation; N^1^: No active stimulation during retention; NA, not applicable; NA^1^: Moxibustion was used as a treatment; No., number; NR, not reported; NR^1^: Duration was reported but frequency was not reported; STRICTA: Standards for Reporting Interventions in Clinic

**Supplementary Table 3.** **Characteristics of the included studies.**

| **Study ID** | **Study design** | **Sample size (M:F);**  **Mean age (y)** | | **Type of NDD** | **Diagnostic criteria of**  **sleep disturbance** | **Duration of sleep disturbance (y)** | **Interventions** | | **CSHQ evaluation subscale** | **Sleep-associated outcomes** | **Intergroup comparison** | **Adverse events (number)** |
| --- | --- | --- | --- | --- | --- | --- | --- | --- | --- | --- | --- | --- |
|  |  | **Treatment** | **Control** |  |  |  | **Treatment** | **Control** |  |  |  |  |
| Mohammadi 2025^39^ | AP+CT(WM) vs. sham-AP+CT(WM) | 23(15:8);  7.87 ± 1.42 | 22(16:6);  9.09 ± 1.27 | ADHD | CSHQ total score ≥ 41 | NR | Auricular AP (5 sessions/wk for 4 weeks; 3 times/session, 3 min/time; 20 sessions total)  +(C) | Sham-AP (only the adhesives without the Vaccaria seeds)  +WM (risperidone, methylphenidate, atomoxetine, or methylphenidate plus risperidone) | Eight | 1. CSHQ total score  1) Bedtime resistance  2) Sleep latency  3) Sleep anxiety  4) Sleep duration  5) Nighttime awakenings  6) Sleep-disordered breathing  7) Parasomnias  8) Daytime sleepiness | 1. T<C+  1) T<C+  2) T<C+  3) T<C+  4) T<C+  5) T<C+  6) N.S  7) T<C+  8) N.S | NR |
| Liu 2024^40^ | AT+CT(with WM) vs. CT  (with WM) | 50(29:21);  9.0 ± 2.0 | 50(27:23);  8.0 ± 2.0 | CP (spastic) | ICD-11 (sleep disorder), CSHQ total score ≥ 41 | NR | AT (5 sessions/wk for 12 wks; 30 min/session; 60 sessions total)+(C) | CT (comprehensive rehabilitation training such as exercise, speech, occupational therapy, educational rehabilitation, family care, dietary guidance, sleep behavior education therapy, and low-frequency repetitive transcranial magnetic stimulation, 20 min/session, once daily, 5 sessions/wk for 12 wks)  +WM(9,000 IU of mouse nerve growth factor (Wuhan Haite Biopharmaceutical Co., Ltd., S20060051), intramuscularly once daily, 10 days/mon for 3 mons) | Eight | 1. The Pittsburgh Sleep Quality Index  2. CSHQ total score | 1. T<C*  2. T<C* | None |
| Yu 2024^24^ | AT+CT vs. CT | 26(22:8);  5.53 ± 2.61 | 27(21:9);  4.53 ± 2.27 | CP | ICSD (sleep disorder) | NR | AT (3 sessions/wk for 12 wks; 30 min/session; 36 sessions total)+(C) | CT (sleep behavior management, sleep health education for 12 wks) | NA | 1. PSG  1) Sleep efficiency  2) Sleep latency  3) Number of nighttime awakenings | 1.  1) T>C*  2) NR  3) T<C* | NR |
| Ma 2023^25^ | Moxibustion+CT vs. CT | 36(19:17);  7.88 ± 3.24 | 36(22:14);  7.55 ± 3.44 | CP (spastic) | CSHQ total score ≥ 45 | NR | Moxibustion (6 sessions/wk for 6 mons; 20 min/session; 144 sessions total)+(C) | CT (multimedia sensory integration training, goal-oriented training, 30 mins each, once daily, 6 sessions/wk for 6 mons) | Eight | 1. TER  2. CSHQ total score  1) Bedtime resistance  2) Sleep latency  3) Sleep anxiety  4) Sleep duration  5) Nighttime awakenings  6) Sleep-disordered breathing  7) Parasomnias  8) Daytime sleepiness  3. PSG  1) Total sleep time  2) Sleep latency  3) Number of nighttime awakenings | 1. T>C*  2. T<C*  1) N.S  2) T<C*  3) N.S  4) N.S  5) N.S  6) N.S  7) N.S  8) N.S  3.  1) T>C*  2) T>C*  3) T>C* | NR |
| Hu 2023^26^ | AT+AP+CT vs. CT | 32(20:12);  6.0 ± 3.0 | 32(17:15);  6.0 ± 2.0 | ASD | DSM-5 (sleep disorder) | NR | AT (3 sessions/wk for 12 wks; 30 min/session; 36 sessions total)  +Auricular AP (2 sessions/wk for 12 wks; 9 min/session; 24 sessions total)+(C) | CT (psychological and behavioral therapy, sleep health education, etiological treatment for 12 wks) | Eight | 1. TER  2. CSHQ total score  3. PSG  1) Total sleep time  2) Sleep latency  3) Number of nighttime awakenings  4) Light sleep time  5) Deep sleep time  6) REM sleep time | 1. T>C*  2. T<C*  3.  1) T>C*  2) NR  3) N.S  4) T>C*  5) T>C*  6) T>C* | NR |
| Zhu 2023^27^ | AT+CT vs. CT | 36(28:8);  3.77 ± 0.73 | 36(26:10);  3.82 ± 0.71 | ASD | CSHQ total score ≥ 41 | NR | AT (6 sessions/wk for 12 wks; 72 sessions total)+(C) | CT (behavioral therapy, gradual extinction method, sensory integration training, active bedtime program and music therapy, etc, 30 min/day, 6 sessions/wk for 24 wks) | Eight | 1. TER  2. CSHQ total score  1) Bedtime resistance  2) Sleep onset delay  3) Sleep anxiety  4) Sleep duration  5) Nighttime awakenings  6) Sleep-disordered breathing  7) Parasomnias  8) Daytime sleepiness | 1. T>C*  2. T<C*  1) T<C*  2) T<C*  3) N.S  4) T<C*  5) T<C*  6) T<C*  7) T<C*  8) T<C* | NR |
| Huang 2022^28^ | AT+AP+CT vs. CT | 50(26:24);  6.58 ± 2.03 | 50(27:23);  6.89 ± 2.05 | CP | CCMD-3 (sleep disorder) | T: 8.42 ± 2.24 mons; C: 8.54 ± 2.31 mons | AT (3 sessions/wk for 2 wks; 30 min/session; 6 sessions total)  +Auricular AP (3 sessions/wk for 2 wks; 20 s/acupoint; 6 sessions total)+(C) | CT (dietary care, medication guidance, etc., for 2 wks) | Eight | 1. TER  2. CSHQ total scores | 1. T>C+  2. T<C+ | NR |
| Xu 2022^29^ | PN+CT vs. CT | 50(40:10);  3.98 ± 1.00 | 50(39:11);  3.91 ± 0.93 | ASD | CSHQ total score ≥ 54 | NR | Auricular PN (once daily for 30 days; 2-4 hours/session; 30 sessions total)  +(C) | CT (sleep hygiene guidance for 30 days) | Five | 1. CSHQ total scores  1) Bedtime habits  2) Morning waking habits  3) Sleeping behavior  4) Nighttime awakenings  5) Daytime sleepiness  2. CSHQ-associated sleep metrics  1) Daily sleep time  2) Weekend sleep time  3) Average sleep time (min) | 1. T<C+  1) N.S  2) T<C*  3) T<C*  4) N.S  5) T<C*  2.  1) N.S  2) N.S  3) N.S | None |
| Sa 2022^30^ | Moxibustion+CT vs. CT | 30(20:10);  6.95 ± 2.24 | 30(18:12);  6.91 ± 2.34 | CP | Encyclopedia of Mongolian Studies (sleep disorder) | T: 0.84 ± 0.39; C: 0.81 ± 0.40 | Moxibustion (once daily for 3 wks; 30 min/session; 21 sessions total)+(C) | CT (gross motor training (40 min/day), fine motor training (30 min/day), speech training (30 min/day), once daily for 3 wks) | NR | 1. TER  2. CSHQ | 1. T>C*  2. NR | NR |
| Zhang 2022^31^ | AT+CT vs. CT | 28(18:10);  8.43 ± 1.83 | 27(16:11);  8.26 ± 1.51 | ASD | CSHQ total score ≥ 41 | T: 5.11 ± 1.50; C: 5.07 ± 1.54 | AT (3 sessions/wk for 6 mons; 30 min/session; 72 sessions total)+(C) | CT (sensory integration training, 2 hours/time, 3 sessions/wk for 6 mons) | Eight | 1. CSHQ total score  1) Bedtime resistance  2) Sleep onset delay  3) Sleep anxiety  4) Sleep duration  5) Nighttime awakenings  6) Sleep-disordered breathing  7) Parasomnias  8) Daytime sleepiness | 2. T<C*  1) T<C*  2) T<C*  3) T<C*  4) T<C*  5) T<C*  6) N.S  7) N.S  8) T<C* | NR |
| Li 2022^32^ | AT+CT vs. CT | 15(10:5);  3.79 ± 1.24 | 15(8:7);  4.03 ± 0.91 | ASD | CSHQ total score > 54 | T: 17.13 ± 4.29 mons; C: 18.73 ± 3.73 mons | AT (6 sessions/wk, 12 sessions/mon (2 wks of treatment, 15 days of rest) for 3 mon; 30 min/session; 36 sessions total)+(C) | CT (extinction method, sleep hygiene, positive reinforcement, sleep restriction, time therapy, stimulus fading strategy, prompt method, scheduled awakening strategy, health education for 3 mons) | Five | 1. CSHQ total score  1) Bedtime habits  2) Morning waking habits  3) Nighttime awakenings  4) Sleep behavior  5) Daytime sleepiness  2. CSHQ-associated sleep metric  1) Average sleep time (min) | 1. T<C*  1) N.S  2) N.S  3) N.S  4) T<C*  5) N.S  2. T>C* | None |
| Dang 2022^33^ | AT+AP+CT(with WM) vs. CT(with WM) | 25(11:14);  4.87 ± 3.59 | 25(13:12);  5.12 ± 2.68 | CP | CSHQ total score > 48 | T: 2.52 ± 1.34; C: 2.45 ± 1.42 | AT (for 40 days; 30 min/session; frequency NR)  +auricular AP (3 sessions/day for 40 days; 2-3 min/session); 120 sessions total)+(C) | CT (behavioral therapy including sleep health education and insomnia stimulation control, 1.5 hours/day, 5 sessions/wk for 40 days)  +WM (antihistamines, sedatives, hypnotics, and antidepressants were selected according to the condition, for 40 days) | Eight | 1. TER  2. CSHQ total score  1) Sleep resistance  2) Sleep onset delay  3) Sleep anxiety  4) Sleep duration  5) Nighttime awakenings  6) Sleep-disordered breathing  7) Parasomnias  8) Daytime sleepiness  3. CSHQ-associated sleep metric  1) Total sleep time | 1. T>C*  2. T<C*  1) T<C*  2) T<C*  3) T<C*  4) T<C*  5) T<C*  6) T<C*  7) T<C*  8) T<C*  3. T>C* | NR |
| Huang 2021^34^ | AT+PN+CT vs. CT | 25(NR);  NR | 25(NR);  NR | ASD (mild to moderate ASD) | CSHQ total score > 41 | NR | AT (5 sessions/wk for 12 wks; min per session NR; 60 sessions total)  +PN (3 sessions/wk for 12 wks; 24 hours/session; 36 sessions total)+(C) | CT (ABA and TEACCH, 2-4 hours/day for 3 mons) | Eight | 1. CSHQ total score  1) Sleep resistance  2) Sleep onset delay  3) Sleep anxiety  4) Sleep duration  5) Nighttime awakenings  6) Sleep-disordered breathing  7) Parasomnias  8) Daytime sleepiness | 1. T<C*  1) T<C*  2) N.S  3) T<C*  4) T<C*  5) T<C*  6) N.S  7) N.S  8) T<C* | NR |
| Li 2021^35^ | PN+CT vs. CT | 41(30:11);  4.92 ± 1.06 | 41(32:9);  4.97 ± 1.11 | ASD | CSHQ total score > 54 | T: 8.17 ± 1.14 mons; C: 8.13 ± 1.26 mons | Auricular PN (7 sessions/10 days (once daily for a wk, and rest 3 days) for 30 days; 2-4 hours/session; 21 sessions total)+(C) | CT (sensory integration training, sensory-motor activities, psychological counseling, language intervention, dietary intervention and sleep intervention for 1 mon) | Eight | 1. TER  2. CSHQ total score | 1. T>C*  2. T<C* | NR |
| Yu 2020^36^ | PN+CT vs. CT | 30(27:3); 49.92 ± 8.02 mons | 30(26:4); 49.75 ± 10.82 mons | ASD | CSHQ total score ≥ 54 | T: 8.21 ± 1.39 mons; C: 8.16 ± 1.43 mons | Auricular PN (once daily for 30 days; 2-4 hours/session; 30 sessions total)+(C) | CT (psychological behavioral treatment, sleep health education for 30 days) | Five | 1. TER  2. CSHQ total score  1) Bedtime habits  2) Sleep behavior  3) Nighttime awakenings  4) Morning waking habits  5) Daytime sleepiness | 1. T>C*  2. T<C*  1) T<C*  2) T<C*  3) N.S  4) T<C*  5) T<C* | T: Local discomfort sensation (1); C: None |
| Zeng 2015^37^ | AT+CT vs. CT | 30(13:17); 5.0 ± 1.0; Intelligence Quotient 53.50±9.27 | 30(16:14); 5.0 ± 1.0; Intelligence Quotient 51.67±7.65 | Intellectual disability | PSG  (sleep efficiency <90%) (sleep disorder) | NR | AT (3 sessions/wk for 12 wks; 30 min/session; 36 sessions total)+(C) | CT (psychological behavior and etiological intervention including sleep hygiene and health education, family training guidance (once daily, 45 min/session), sensory integration training (once daily, 1 hour/session) for 12 wks) | NA | 1. TER  2. PSG  1) Sleep latency  2) REM sleep  3) Sleep efficiency | 1. T>C*  2.  1) T>C*  2) T>C*  3) T>C* | NR |
| Chen 2014^38^ | TEAS+CT vs. CT | 30 | 30 | ASD | CCMD-3 (sleep disorder) | NR | TEAS (5 sessions/wk for 2 mons; 30 min/session; 40 sessions total) +(C) | CT (cognitive behavioral rehabilitation training, once daily for 2 mons) | Eight | 1. TER  2. CSHQ total score  1) Bedtime resistance  2) Sleep onset delay  3) Sleep anxiety  4) Sleep duration  5) Nighttime awakenings  6) Sleep-disordered breathing  7) Parasomnias  8) Daytime sleepiness | 1. T>C+  2. T<C+  1) N.S  2) T>C+  3) T<C*  4) N.S  5) T<C+  6) T<C+  7) T<C+  8) T<C+ | NR |
|  |  | 60(36:24); 5.06 ± 1.32 | |  |  |  |  |  |  |  |  |  |

Notes: *, p <0.05; +, p <0.01; N.S, p >0.05; ABA, applied behavior analysis; ADHD, attention-deficit hyperactivity disorder; AP, acupoint pressure; ASD, autism spectrum disorder; AT, acupuncture; C, control group; CCMD-3, Third Edition of the Chinese Classification and Diagnostic Criteria for Mental Disorders; CP, cerebral palsy; CSHQ, Children's Sleep Habits Questionnaire; CT, conventional treatment; DSM-5, Diagnostic and Statistical Manual of Mental Disorders, 5th Edition; ICD-11: International Classification of Diseases 11th Revision; ICSD, International Classification of Sleep Disorders; PSG, polysomnography; PN, press needle; REM, rapid eye movement; s, second; TEACCH, Treatment and Education of Autistic and Communication related handicapped Children; TEAS, transcutaneous electric acupoint stimulation; TER, total effective rate; min, minute; mon, month; Moxa, moxibustion; NA, not applicable; NR, not reported; NDD, neurodevelopmental disease; vs., versus; wk, week; WM, western medicine; y, year.

**Supplementary Table 4.** **Detailed information on acupuncture-related treatment groups.**

| **Study ID** | **AT type**  **(Protocol)** | **Acupoints** | **Depth of needle insertion** | **Needle manipulation** | **Type of needle/moxibustion (Diameter, length, manufacturer)** | **Number of treatment sessions** | **Frequency** | **Retention time** | **Treatment duration** |
| --- | --- | --- | --- | --- | --- | --- | --- | --- | --- |
| Mohammadi 2025^39^ | AP  (Standardized) | Shenmen (TF4), Sympathetic (AH6a), Subcortex (AT4), Heart (CO15), Endocrine (CO18) (unilateral, alternating stimulation of both ears once a week) | NR | Manual (press 3 times/day, 3 minutes/time) | Vaccaria seeds (DongBang Co., Ltd., Korea) | 20 | 5 times/wk | 5 days | 4 wks |
| Liu 2024^40^ | AT  (Standardized) | Benshen (GB13), Ganshu (BL 18), Shenshu (BL 23), Shenmai (BL 62), Zhaohai (KI 6) (bilateral), 2 cun above Erjian (EX-HN6), 1 cun anterior and posterior of Erjian (EX-HN6), Fengchi (GB20), Wangu (GB12), Tianzhu (BL10) (no information about side), Shenting (GV24), Sishencong (EX-HN1), | Ganshu (BL 18), Shenshu (BL 23): 5–15 mm; Shenmai (BL 62), Zhaohai (KI 6): 10–30 mm | Manual (lifting–thrusting and twirling–rotating manipulation to obtain qi) | 0.30 mm × 25 mm, disposable sterile needles | 60 | 5 times/wk | 30 min | 12 wks |
| Yu 2024^24^ | AT  (Standardized) | Baihui (GV20), Shenting (GV24), Anmian (EX-HN18), Tianzhu (BL10), Shenmen (HT7), Neiguan (PC6) (no information about side) | NR  -Scalp acupoints: obliquely at an angle of 15°–30° | Manual (lifting-thrusting and twirling manipulation to obtain qi) | NR | 36 | 3 times/wk | 30 min | 12 wks |
| Ma 2023^25^ | Moxibustion  (Standardized) | Fuyang (BL59) (bilateral), Shenting (GV24), Dazhui (GV14), Zhiyang (GV9), Mingmen (GV4) | NA (transcutaneous) | NA | Indirect moxibustion device, Zhongshan Jiaxin Medical Instrument Co., Ltd, CAJ-2000 model | 144 | 6 times/wk | 20 min,  38–40 °C | 6 mons |
| Hu 2023^26^ | AT+AP  (Semi-standardized) | AT: Baihui (GV20), Yintang (EX-HN3), Anmian (EX-HN18), Shenmen (HT7), Zusanli (ST36), Sanyinjiao (SP6). Add the following acupoints according to the symptoms.  -Dual deficiency of the heart-spleen: +Xinshu (BL15), Pishu (BL20).  -Heart-gallbladder qi deficiency: +Xinshu (BL15), Danshu (BL19), Yanglingquan (GB34).  -Stomach disharmony: +Zhongwan (CV12).  -Liver fire flaming upward: +Ganshu (BL18), Daling (PC7), Xingjian (LR2).  -Yin deficiency with effulgent fire: +Xinshu (BL15), Shenshu (BL23), Zhaohai (KI6).  ; AP: Shenmen (TF4), Heart (CO15), Kidney (CO10), Liver (CO12), Spleen (CO13), Sympathetic (AH6a), Subcortex (AT4) (no information about side) | AT: 0.5 cun,  -Limbs acupoints: perpendicularly; Scalp and transport points: obliquely | AT: Manual (neutral supplementation and draining manipulation); AP: Manual (press 3 times/day, 3 minutes/time) | AT: 0.25 mm × 25 mm; AP: Press with Vaccaria seeds | AT: 36;  AP: 24 | AT: 3 times/wk; AP: 2 times/wk (once every 3 days) | AT: 30 min (scalp only, no retention on the others);  AP: 72 hrs | 12 wks |
| Zhu 2023^27^ | AT  (Standardized) | Xinshu (BL15), Pishu (BL20), Shenshu (BL23), Ganshu (BL18), Weishu (BL21), Tianshu (ST25), Juque (CV14), Guanyuan (CV4), Shanzhong (CV17), Zhongwan (CV12) (no information about side) | The depth of needle insertion depends on the child’s fatness.  -Chest and back: obliquely; Abdomen and waist: perpendicularly | Manual (rapid insertion and withdrawal manipulation) | 0.30 mm × 30 mm, disposable needles | 72 | 6 times/wk, | No retention | 12 wks |
| Huang 2022^28^ | AT+AP  (Semi-standardized) | AT: Naokong (GB19), Benshen (GB13) (bilateral), Sishencong (EX-HN1), Naohu (GV17), Shenting (GV24), Neiguan (PC6), Shenmen (HT7), Laogong (PC8), Yongquan (KI1), Anmian (EX-HN18), 2 cun above Erjian (EX-HN6), 1 cun anterior and posterior of Erjian (EX-HN6). Add the following acupoints according to the symptoms.  -Physical disability: +Shousanli (LI10), Zusanli (ST36), Hegu (LI4), Quchi (LI11), Feiyang (BL58), Yanglingquan (GB34)  -Language disorder: +Shanglianquan (upper CV23), Shanglianquan (upper CV23), 0.8 cun left and right of Shanglianquan (upper CV23) (no information about side); AP: Shenmen (TF4), Sympathetic (AH6a), Endocrine (CO18), Subcortex (AT4), Heart (CO15) (unilateral, alternating stimulation of both ears). Add the following acupoints according to the symptoms.  -Dual deficiency of the heart-spleen: +Spleen (CO13)  -Liver depression and qi stagnation: +Liver (CO12), Gallbladder (CO11)  -Non-interaction between the heart and kidney: +Kidney (CO10) | NR | AT: Manual (needling manipulation to obtain qi); AP: Manual (press with a probe) | AT: 0.30 mm × 25 mm, Huatuo brand disposable 30 gauge filiform needles; AP: press with probe | AT: 6;  AP: 6 | 3 times/wk | AT: 30 min;  AP: 20 s/acupoint | 2 wks |
| Xu 2022^29^ | PN  (Standardized) | Shenmen (TF4), Heart (CO15), Liver (CO12), Subcortex (AT4) (no information about side) | NR | No active stimulation | Disposable needles, Seirin Pyonex, Japan Co., Ltd., National Medical Device Injection No. 0162271259 | 30 | Once daily | 2–4 hrs | 30 days |
| Sa 2022^30^ | Moxibustion  (Standardized) | Dinghui (GV20), Heyi Acupoint (on the 1st thoracic vertebrae spinous process), Mingmai Acupoint (between the fifth and sixth thoracic vertebrae spinous processes), Xin Acupoint (between the sixth and seventh thoracic vertebrae spinous processes), Shanzhong (CV17) | NA (transcutaneous) | NA | Mongolian moxibustion (consists of *Aquilariae Lignum Resinatum*) | 21 | Once daily | 30 min | 3 wks |
| Zhang 2022^31^ | AT  (Standardized) | Zusanli (ST36), Quchi (LI11), Tianshu (ST25), Shangjuxu (ST37) (bilateral), Qihai (CV6), Guanyuan (CV4), Baihui (GV20), Yintang (EX-HN3) | NR | Manual (smooth needling manipulation) | NR | 72 | 3 times/wk | 30 min | 6 mons |
| Li 2022^32^ | AT  (Standardized) | The Fuxiang head, Fuzang Upper Jiao (projection area of ​​the frontal lobe on the scalp), thinking (located in the projection area of ​​the hypothalamic preoptic area on the forehead skin), signal (projection area of ​​the posterior part of the superior temporal gyrus on the scalp), and memory (projection area of ​​the inferior parietal lobule on the scalp) (no information about side) | NR | Manual (periosteal tapping needling manipulation, once every 15 mins) | 0.30 mm × 16 mm, disposable needles, Ma'anshan Bond Medical Equipment Co., Ltd. | 36 | 12times/4wk (6 times/wk for 2 wks, then 15 days rest) | 30 min | 12 wks |
| Dang 2022^33^ | AT+AP  (Semi-standardized) | AT: Sanyinjiao (SP6), Zhaohai (KI6), Shenmai (BL62), Anmian (EX-HN18), Shenting (GV24), Shenmen (HT7), Baihui (GV20), Sishencong (EX-HN1) (no information about side); AP: 2-3 positive reaction points on the cymba conchae and cavum conchae (unilateral, alternating stimulation of both ears) | NR | AT: NR; AP: Manual (press 3 times/day, 1 min/(acupoint⋅time)) | AT: Disposable filiform needles; AP: apply Vaccaria seeds with 5 mm × 5 mm medical tape | AT: NR;  AP: 120 | NR | AT: 30 min;  AP: NR | 40 days |
| Huang 2021^34^ | AT+PN  (Semi-standardized) | AT: Benshen (GB13), 2 cun above Erjian (EX-HN6), 1 cun anterior and posterior of Erjian (EX-HN6) (bilateral), Sishencong (EX-HN1), Shenting (GV24); PN: Yangbai (GB14) (bilateral), Yintang (EX-HN3). Add the following acupoints according to the symptoms.  -Liver depression and qi stagnation: +Hegu (LI4), Taichong (LR3).  -Effulgent heart-liver fire: +Shaofu (HT8), Xingjian (LR2)  -Phlegm clouding the pericardium: +Fenglung (ST40), Daling (PC7).  -Kidney essence insufficiency: +Taixi (KI3). | AT: 0.5–0.8 cun | AT: Manual (twirling manipulation); PN: No active stimulation | AT: 0.30 mm × 30 mm, Jiajian brand disposable stainless steel needle; PN: 0.13 mm × 2.0 mm, disposable stainless steel needle, Wuxi Jiajian Medical Equipment Co., Ltd. | AT: 60;  PN: 36 | AT: 5 times/wk; PN: 3 times/wk  (maintain 24 hrs and rest 24 hrs) | AT: NR;  PN: 24 hrs | 12 wks |
| Li 2021^35^ | PN  (Semi-standardized) | Select 6 to 8 acupoints below: Lung (CO14), Diaphragm (CO16), Subcortex (AT4), Endocrine (CO18), Intestine (CO9), Heart (CO15), Shenmen (TF4), Liver (CO12) (unilateral, alternating stimulation of both ears) | 0.60 mm, perpendicularly | Manual (twisting and pressing manipulation) | 0. 20 mm × 0. 60 mm, disposable sterilized Qingling needle, Japan Seirin Co., Ltd. | 21 | 7 times/10 days (once daily for a wk, then rest 3 days) | 2–4 hrs | 30 days |
| Yu 2020^36^ | PN  (Standardized) | Shenmen (TF4), Heart (CO15), Liver (CO12), Subcortex (AT4) (no information about side) | NR | Manual (press until the ear turns red) | Disposable needles, Japan Co., Ltd., National Medical Device Injection No. 20162271259 | 30 | Once daily | 2–4 hr | 30 days |
| Zeng 2015^37^ | AT  (Semi-standardized) | Fengchi (GB20), Anmian (EX-HN18), Shenmen (HT7), Neiguan (PC6), Zusanli (ST36), Sanyinjiao (SP6), Taichong (LR3), Fenglong (ST40) (bilateral), Baihui (GV20), Sishencong (EX-HN1), Yintang (EX-HN3). Add or subtract acupoints according to symptoms | 0.5 cun  -Scalp acupoints: transversely; Back acupoints: obliquely; Body acupoints: perpendicularly | Manual (twirling and lifting-thrusting manipulation) | 0.25 mm × 25 mm, filiform needles, Suzhou Medical Supplies Factory Co., Ltd. | 36 | 3 times/wk | 30 min (scalp only, no retention on the others) | 12 wks |
| Chen 2014^38^ | TEAS  (Standardized) | Baihui (GV20), Shenting (GV24), Yintang (EX-HN3), Shenmen (HT7), Anmian (EX-HN18), Sanyinjiao (SP6), Taixi (KI3) (Unilateral) | NA (transcutaneous) | Electrical (4–6 mA, 2/100 Hz double-frequency modulation, continuous stimulation) | The HANS® acupoint nerve stimulator | 40 | 5 times/wk | 30 min | 2 mons |

Notes: AT, acupuncture; AP, acupressure; hr, hours; mon, month; min, minutes; NR, not reported; NA, not applicable; PN, press needle; s, second; TEAS: transcutaneous electric acupoint stimulator; wk, week.
