# Supplementary figures and images for: Efficacy of acupuncture-related treatment for sleep disturbances in children with neurodevelopmental disorders: a systematic review and meta-analysis
Source: Front Psychiatry. 2026 Jan 13;16:1670438. doi: 10.3389/fpsyt.2025.1670438 (PMC12835266; doi:10.3389/fpsyt.2025.1670438)

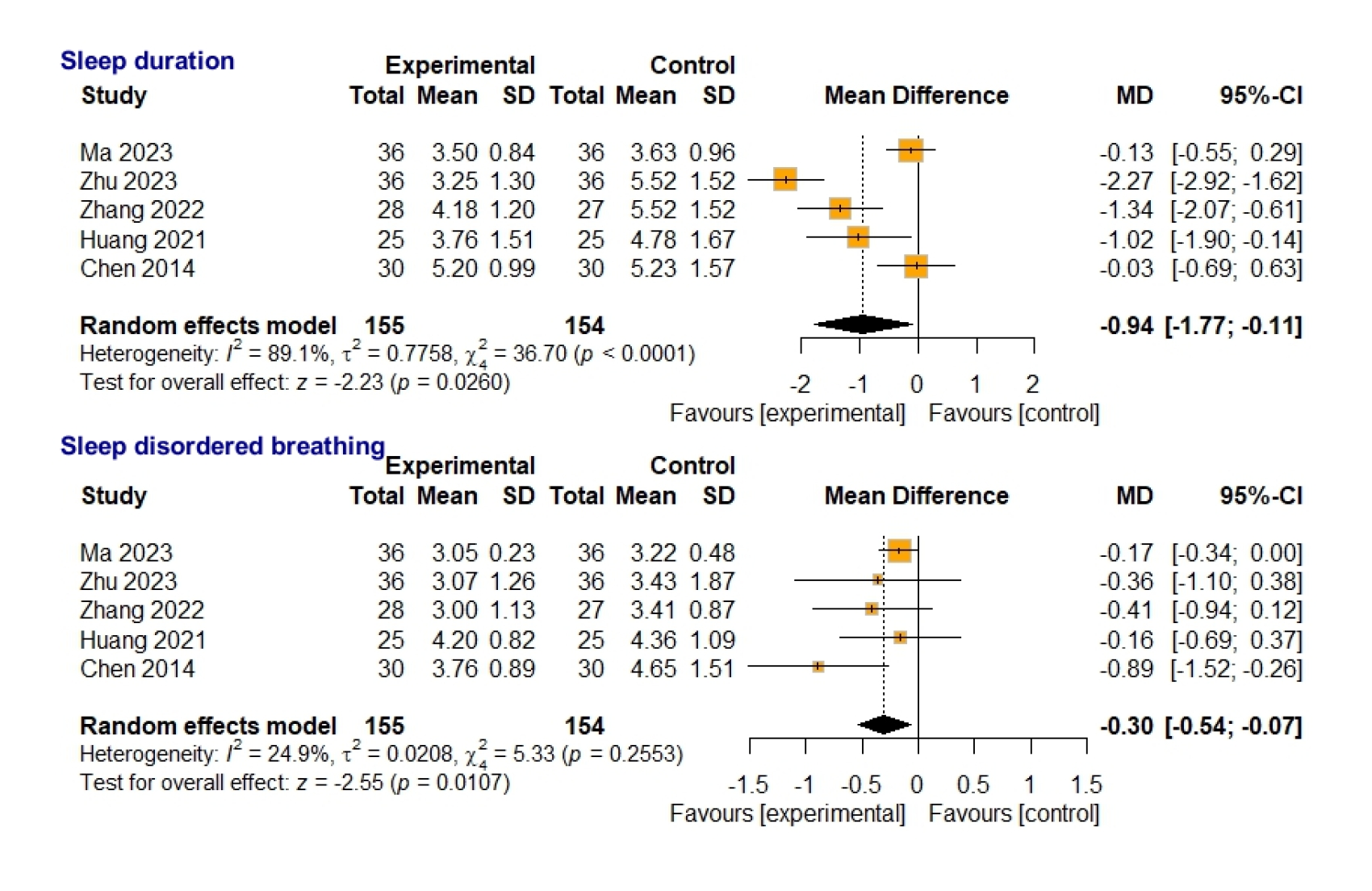

Supplement: Supplementary Figure 1 — Forest plot comparing acupuncture-related treatment plus CT vs. CT on CSHQ 8-subscale scores. AT, acupuncture; CT, conventional treatment; CSHQ, Children’s Sleep Habits Questionnaire [file Presentation1.zip › Supplementary figure 1.TIFF]

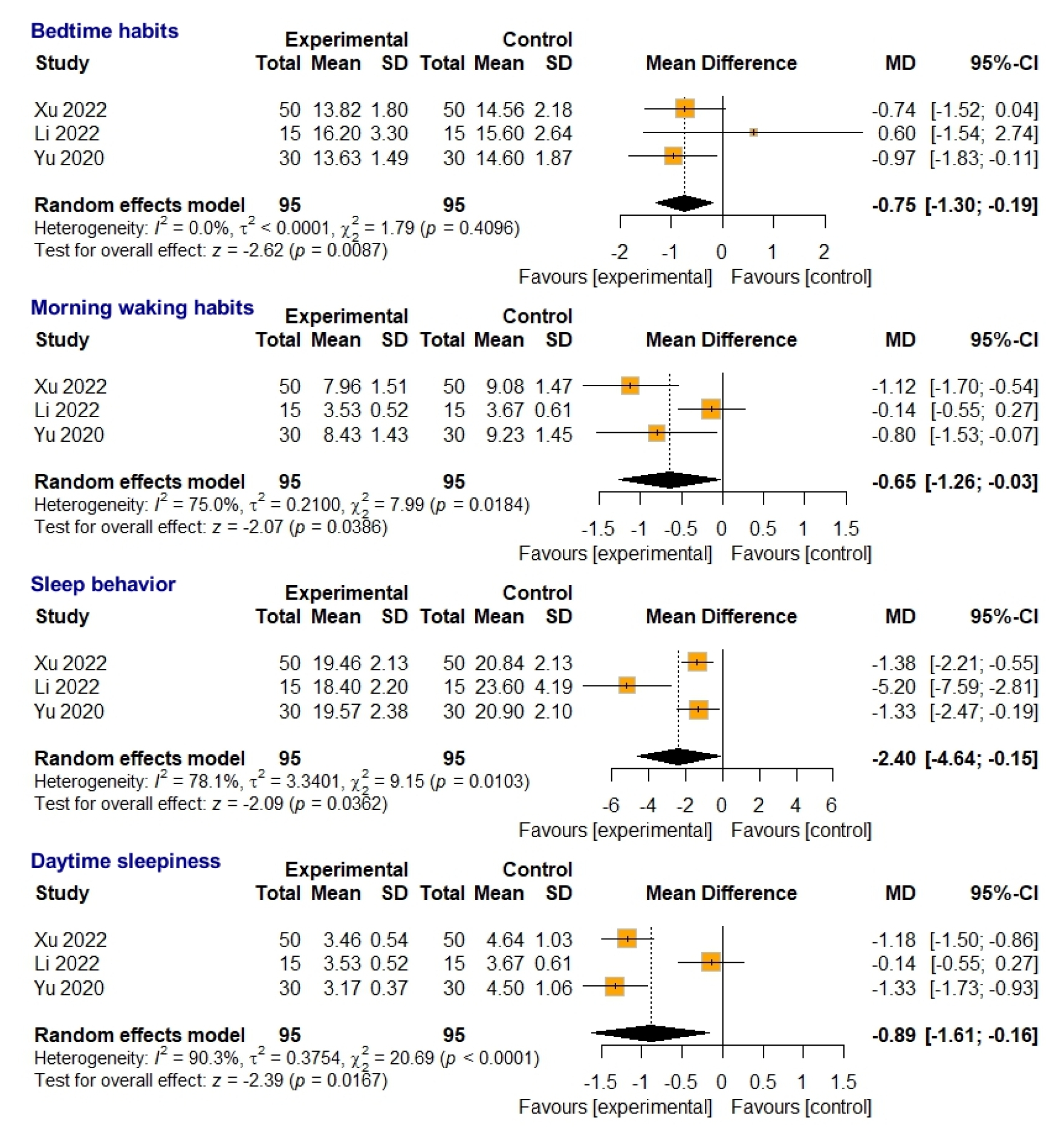

Supplement: Supplementary Figure 1 — Forest plot comparing acupuncture-related treatment plus CT vs. CT on CSHQ 8-subscale scores. AT, acupuncture; CT, conventional treatment; CSHQ, Children’s Sleep Habits Questionnaire [file Presentation1.zip › Supplementary figure 2.TIFF]

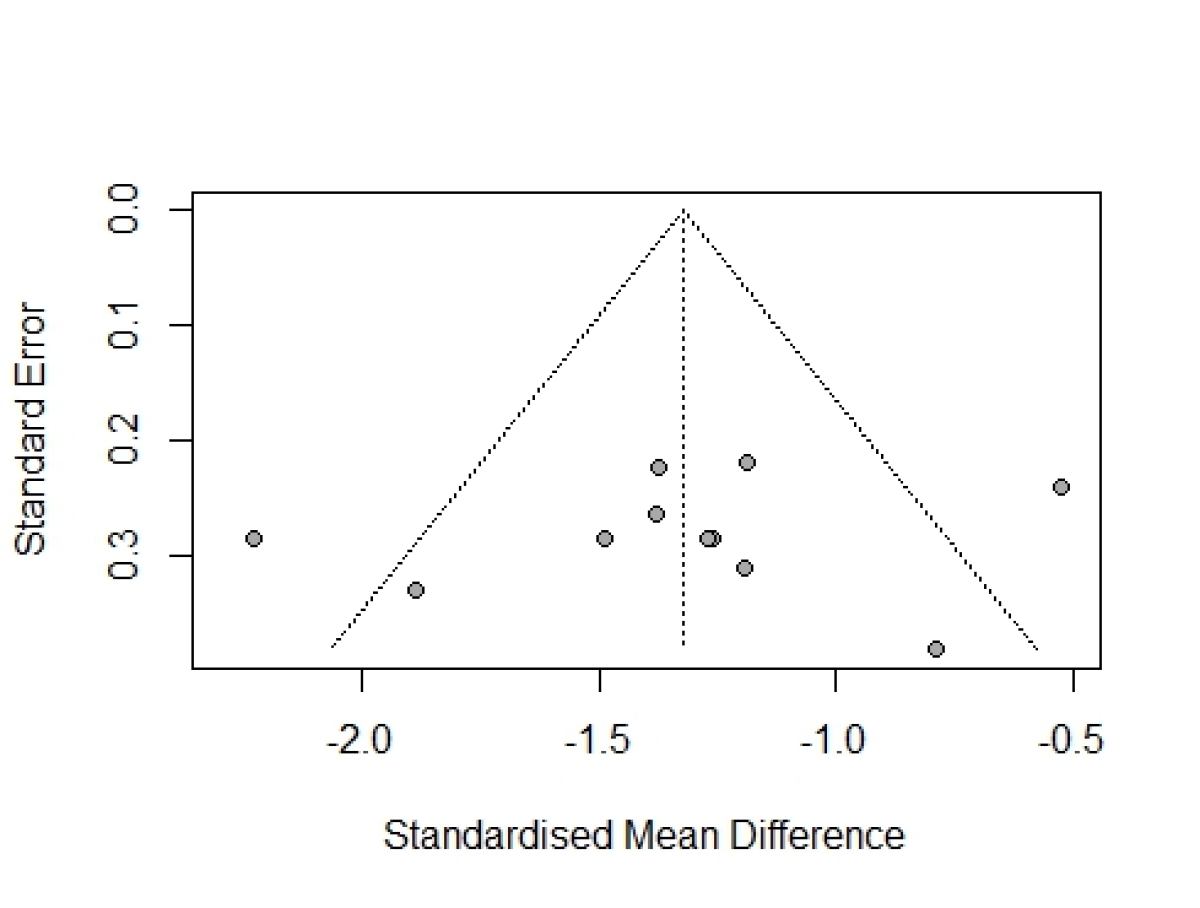

Supplement: Supplementary Figure 1 — Forest plot comparing acupuncture-related treatment plus CT vs. CT on CSHQ 8-subscale scores. AT, acupuncture; CT, conventional treatment; CSHQ, Children’s Sleep Habits Questionnaire [file Presentation1.zip › Supplementary figure 3.TIFF]
